# ZFHX3 acts as a tumor suppressor in prostate cancer by targeting FTO-mediated m^6^A demethylation

**DOI:** 10.1038/s41420-024-02060-w

**Published:** 2024-06-13

**Authors:** Qingxia Hu, Junling Yin, Sijie Zhao, Yibo Wang, Ruxue Shi, Keqiang Yan, Shuhong Huang

**Affiliations:** 1 School of Clinical and Basic Medicine, Shandong First Medical University & Shandong Academy of Medical Sciences, Jinan, Shandong China; 2 Shandong Provincial Hospital of Shandong First Medical University & Shandong Academy of Medical Sciences, Jinan, Shandong China; 3 Medical Science and Technology Innovation Center, Shandong First Medical University & Shandong Academy of Medical Sciences, Jinan, Shandong China; 4 Department of Urology, Qilu Hospital of Shandong University, Jinan, 250012 Shandong China

**Keywords:** Prostate cancer, Molecular biology

## Abstract

Zinc-finger homeobox 3 (ZFHX3, also known as ATBF1) suppresses prostatic tumorigenesis. ZFHX3 is frequently found to have numerous deletions in human prostate cancer (PCa). However, the underlying molecular function of ZFHX3 during prostatic tumorigenesis is not well understood. *N*^6^-methyladenosine (m^6^A) modification in RNA plays a critical role in the development of cancers; however, the relationship between ZFHX3 and m^6^A modification is largely unknown in PCa. In this study, we found that ZFHX3 knockdown decreased total m^6^A levels through enhancing the transcriptional activity of *FTO* in PCa cells. Importantly, FTO inhibition suppressed cell proliferation and rescued the promoting function of ZFHX3 knockdown on cell proliferation. In vivo, we verified that FTO was upregulated and ZFHX3 was decreased in PCa patients and that a high level of ZFHX3 is indispensable for low FTO expression and is correlated with better patient survival. Through transcriptome sequencing and MeRIP sequencing, we revealed that E2F2 and CDKN2C were the direct targets of FTO-mediated m^6^A modification and ZFXH3 was required for the regulation of FTO on E2F2 and CDKN2C expression. Unexpectedly, we uncovered that ZFHX3 expression was in return regulated by FTO in an m^6^A-dependent way. These findings establish a novel crosstalk mechanism between ZFHX3 and FTO in prostatic tumorigenesis.

## Introduction

Prostate cancer (PCa) is one of the most common cancers and demonstrates a high mortality rate in men [1]. Despite advances in diagnostic methods and the therapeutic landscape, most PCa patients will eventually experience recurrence and distant organ metastasis [2]. Existing therapies may be effective at earlier stages of tumor, but the progression of prostate tumor is unpredictable; as a result, a cure for PCa remains elusive. There is an urgent need to explore substantial molecular mechanisms underlying the progression of PCa.

In eukaryotes, *N*^6^-methyladenosine (m^6^A) is the most common and conserved internal modification in different types of RNA [3, 4]. m^6^A modification is a reversible biological process instigated by methyltransferase complexes (METTL3 [5], METTL14 [6], METTL16 [7], WTAP [8], RBM15 [9], KIAA1429 [10], and ZC3H13 [11], also known as “writers”) and removed by demethylases (FTO [12], ALKBH3 [13], and ALKBH5 [14], also known as “erasers”). The m^6^A-modified RNA can be recognized by m^6^A reader proteins, such as YTHDC1/2 [15, 16] and YTHDF1/2/3 [17, 18]. m^6^A modification is crucial for embryonic development, central nervous system development, and hematopoiesis. In addition, many studies have shown that m^6^A plays important roles in various diseases, including heart failure [19], fatty liver disease [20], and especially human cancers [21–26].

The demethylase FTO reverses the m^6^A modification in RNA to maintain a balanced m^6^A level in cells. In acute myeloid leukemia (AML), much evidence shows that FTO demethylase is highly expressed and performs an oncogenic role in promoting cell proliferation and inhibiting apoptosis [27–29]. In melanoma cells, FTO impairs IFNγ-induced cell death and inhibits cells’ response to the immunotherapy of an anti-PD-1 blockade [30]. The mRNA level of FTO is upregulated in cervical squamous cell carcinoma [31] and enhances chemo–radiotherapy resistance by increasing the expression of β-catenin and ERCC excision repair 1 [32]. In addition, FTO plays an oncogenic role in lung cancer and breast cancer by targeting USP7 and BNIP3, respectively [33, 34]. In addition to the cancers listed above, some evidence suggests that FTO represses the progression of PCa metastasis and inhibits the degradation of chloride intracellular channel 4 mRNA in PCa cells [35]. Little is known about whether there are other functions of FTO in PCa or what the underlying mechanism of m^6^A is in the pathogenesis and progression of PCa.

Zinc-finger homeobox 3 (ZFHX3, also known as ATBF1 for AT-motif binding factor 1) is a transcription factor [36]. ZFHX3 plays important roles in neuronal differentiation and in the regulation of circadian locomotor rhythms in the suprachiasmatic nucleus [37–39]. In mice, ZFHX3 regulates pubertal mammary gland development and modulates the proper lactogenic function in mammary glands [40–42]. ZFHX3 has frequent mutations in human PCas, and most of the mutations induce ZFHX3 function deactivation [43, 44]. In mouse models, *Zfhx3* deletion in mouse prostates causes prostate intraepithelial neoplastic or tumor growth and facilitates prostatic tumorigenesis induced by deletion together with *Pten* [45, 46]. Although the tumor-suppressive role of ZFHX3 has been indicated in previous studies, it is not clear how ZFHX3 exerts a suppressor function in PCa. Our previous study demonstrated that ZFHX3 is essential for ERβ signaling to inhibit cell proliferation through repressing *MYC* expression [47]. This study provides mechanistic evidence to establish ZFHX3 as a tumor suppressor in PCa. However, more research is urgently needed to explore the underlying mechanism in PCa, and we hope to find a novel biomarker that could provide potential targets for PCa therapy in the future.

In this study, we report that FTO is the downstream target of ZFHX3 and is upregulated in prostate tumor tissues compared to normal prostate tissues. Meanwhile, we verify the function of FTO in vitro, which is consistent with the role of FTO in prostate patients. ZFHX3 is important given its effect on FTO-mediated E2F2 and CDKN2C on PCa cell proliferation. More importantly, we found a feedback loop between ZFHX3 and FTO, which implicates FTO as a mediator of the expression of ZFHX3. Thus, our data demonstrate a crosstalk between ZFHX3 and FTO-mediated CDKN2C and E2F2 expression, which has a critical effect on PCa progression and provides insight into clinical biomarkers for the prognosis and therapeutics of PCa.

## Results

### ZFHX3 knockdown decreases the total m^6^A levels in PCa cells

**Fig. 1:**
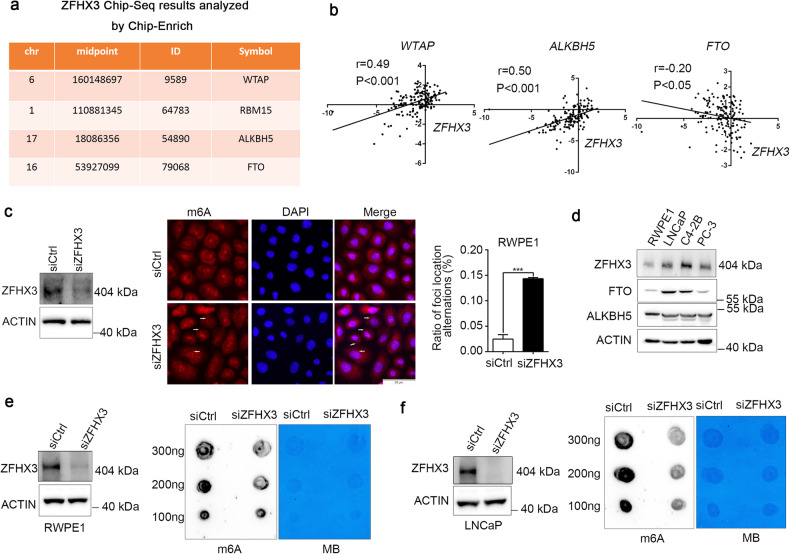
Inhibition of ZFHX3 represses total m^6^A modification in both RWPE1 and LNCaP cell lines. **a** The genes involved in m^6^A modification are shown in the table as candidate targets of ZFHX3 and were analyzed by Chip-Enrich using the ZFHX3 Chip-Seq results. **b** *ZFHX3* correlated with *WTAP*, *FTO*, and *ALKBH5* when using a cluster of PCa patients containing mRNA expression of these genes. **c** The m^6^A level was detected by immunofluorescence in RWPE1 cells after knocking down ZFHX3, and the arrows indicated m^6^A translocating from the nucleus to the cytoplasm. *n* = 3. **d** The expression of ZFHX3, FTO, and ALKBH5 in human prostate epithelial cell lines, was determined by western blot. The efficiency of ZFHX3 knockdown was detected by western blot, and the global m^6^A levels were reduced by ZFHX3 knockdown and detected by RNA dot blot in RWPE1 (**e**) and LNCaP (**f**). *n* = 3. MB (methylene blue) represents the loading control of the RNA samples. ***P < 0.001.

Initially, we analyzed two sets of published ChIP-seq data of ZFHX3 [41, 48] and noted that several critical modulators of m^6^A modification, i.e., WTP, RBM15, ALKBH5, and FTO, are potential target genes of ZFHX3 (Fig. 1a). Further, we found that ZFHX3 has a moderate positive correlation with WTAP and ALKBH5, and a weak negative correlation with FTO in microarray mRNA data from GSE21032 (Fig. 1b). To confirm the relationship between ZFHX3 and m^6^A modification, we knocked down ZFHX3 with siRNA in human immortalized prostate epithelial cell line RWPE1, and found that more m^6^A-modified mRNAs were translocated from the nucleus to the cytoplasm and that signals of m^6^A were diffused in the nucleus (Fig. 1c). To investigate the mechanism underlying the phenotype induced by ZFHX3 deletion, we focused on the function of m^6^A demethylases, including FTO and ALKBH5. We first detected the expression of FTO and ALKBH5 in some PCa cell lines by western blot (Fig. 1d). Compared to nontumorous RWPE1 cells, ZFHX3 and FTO expressed were highly expressed in PCa cell lines LNCaP and C4-2B, whereas ALKBH5 had only moderate expression in PCa cell lines LNCaP, C4-2B, and PC-3. In this study, we chose LNCaP and RWPE1 cell lines to investigate the function of ZFHX3 in m^6^A modification. Next, we tested whether ZFHX3 influenced the global m^6^A level by RNA dot blot. Consistent with the findings in Fig. 1c, ZFHX3 knockdown by siRNA decreased global m^6^A abundance with different RNA concentrations (100, 200, and 300 ng) in LNCaP and RWPE1 cells (Fig. 1e, f). These results suggest that there was a negative correlation between ZFHX3 and FTO; however, the regulatory relationship between ZFHX3 and FTO was still unknown.

### Overexpression of ZFHX3 suppresses PCa proliferation in vivo

**Fig. 2:**
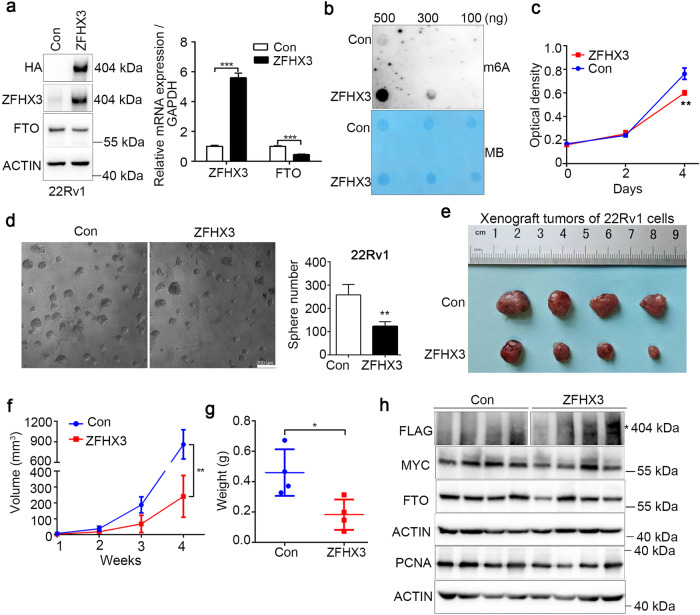
Overexpression of ZFHX3 suppresses tumor growth in 22Rv1 prostate cancer cells. **a** The efficiency of ZFHX3 overexpression was detected by western blot and RT-qPCR. **b** The global m^6^A levels were increased by ZFHX3 overexpression detected by RNA dot blot in 22Rv1 cells. ***P < 0.001. **c** Ectopically expressed ZFHX3 decreased cell proliferation, as measured by the SRB assay. **P < 0.01. **d** ZFHX3 overexpression decreased sphere formation in Matrigel. The data were presented as the average number of spheres with a diameter >75 µm/well. *n* = 3. **e** The NOD-scid mice were sacrificed for the xenografts at 28 days, and the size was indicated by the beside ruler. Tumor volumes were measured at the indicated times (**f**) and isolated tumors were weighted (**g**). *P < 0.05; **P < 0.01. **h** Detection of the indicated molecules in cells stably transfected with control-vector or ZFHX3 by western blot.

To further validate the functional role of ZFHX3 in PCa, we performed a subcutaneous implantation experiment in male NOD-scid mice to explore the effect of ZFHX3 overexpression in PCa progress. We established stable 22Rv1 cell lines expressing either control-vector or Flag-ZFHX3 and subsequent western blot and quantitative real-time PCR (qRT-PCR) analysis revealed the overexpression of ZFHX3 in 22Rv1 cells (Fig. 2a). In addition, overexpression of ZFHX3 enhanced m^6^A modification in 22Rv1 cells (Fig. 2b), which was consistent with the effect of ZFHX3 in RWPE1 and LNCaP cells. SRB assay proved that overexpression of ZFHX3 inhibited cell proliferation (Fig. 2c). Moreover, sphere formation was decreased in ZFHX3 overexpression cells (Fig. 2d). ZFHX3 was knocked down by shRNAs in LNCaP cells and the knockdown promoted sphere formation (Fig. S1). Overexpression of ZFHX3 suppressed the growth of prostate tumors, which was reflected by the tumor size and weight compared with the control tumors (Fig. 2e–g). Western blot assay was used to detect the expression of ZFHX3, MYC, FTO, and PCNA in tumor tissues. In these tumors with ZFHX3 overexpression, MYC and FTO expression was slightly decreased and PCNA expression had no change (Fig. 2h). In addition, as shown in Supplementary Fig. 2, ZFHX3 was not associated with T stage, but both Gleason score and metastasis stage were associated with the expression of ZFHX3. This result indicates that ZFHX3 plays an important role in the progression of PCa, especially in malignant prostate tumor, which is consistent with the previous study about the role of ZFHX3 in prostate tumor [44]. All these results suggest that ZFHX3 plays a pivotal role in suppressing PCa progression in vivo.

### ZFHX3 suppresses acini formation depending on the inhibition of FTO

**Fig. 3:**
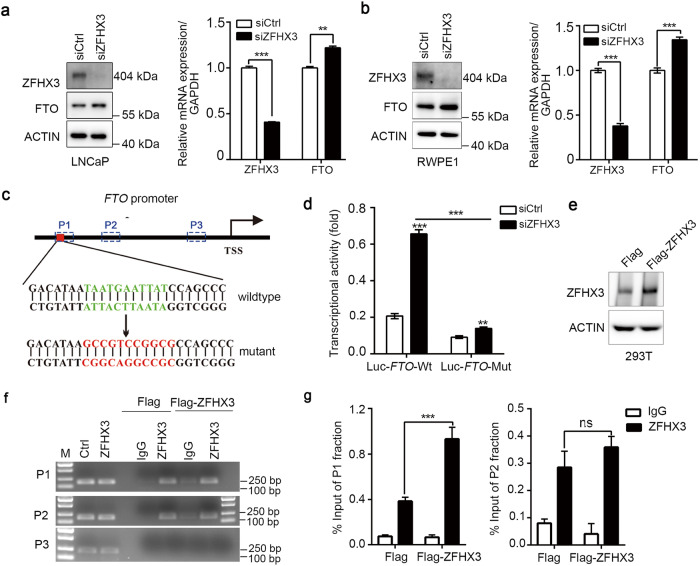
Inhibitory effect of ZFHX3 on acini formation depends on the inhibition of FTO in RWPE1 cells. **a**, **b** Knockdown of ZFHX3 upregulated FTO expression in RWPE1 and LNCaP cells at the protein and mRNA levels, as detected separately by western blot and qRT-PCR. *n* = 3. **c** The wild type and mutant of the *FTO* promoter were constructed, and the critical sequences are shown in the panel. Red rectangles indicate mutant sequences, and blue rectangles indicate the regions within the *FTO* promoter for PCR amplification. **d** ZFHX3 knockdown increased the activity of the wild-type promoter of *FTO*, but the effect on the mutant promoter of *FTO* was partly impaired compared to the wild-type promoter. *n* = 3. **e** Expression plasmids for control-vector (Flag) or Flag-ZFHX3 were transfected in 293T cells and the efficiency was detected by western blot. **f** Detection of ZFHX3-bound *FTO* promoter DNA in ZFHX3-overexpressed cells or control cells was completed using ChIP and regular PCR. **g** Binding of ZFHX3 to *FTO* promoter region 1 and region 2 was found using ChIP and qRT-PCR. *n* = 3. **P < 0.01; ***P < 0.001; ns not significant.

To explore the role of ZFXH3 in FTO expression, we first examined the role of ZFHX3 on FTO expression in LNCaP and RWPE1 cells (Fig. 3a, b) and validated that *ZFHX3* repressed the mRNA and protein expression of FTO. To investigate whether ZFHX3 affected the activity of the *FTO* promoter, we predicted the candidate binding sites of ZFHX3 on *FTO* using the hTFtarget database [49] and constructed the wild type and mutant *FTO* promoters (Fig. 3c). We performed a luciferase promoter assay to assess the role of ZFHX3 in *FTO* transcription (Fig. 3d). We found ZFHX3 knockdown significantly enhanced the activity of the wild-type promoter of *FTO*, which was partly impaired by the mutant promoter. To verify the critical regions of the *FTO* promoter that are bound by ZFHX3, we used ChIP–PCR. Based on the analysis of ChIP-seq data about ZFHX3 from the Cistrome DB online database, we obtained three regions within the *FTO* promoter for PCR amplification that are indicated as blue rectangles in Fig. 3c. In 293T cells, we transfected Flag-ZFHX3 plasmid or Flag-vector (Fig. 3e). ChIP–PCR analyses showed that ZFHX3 bound to the P1 and the P2 regions of the *FTO* promoter but not to the P3 region (Fig. 3f). Meanwhile, ZFHX3 overexpression enhanced the connection between ZFHX3 and the P1 region of the *FTO* promoter (Fig. 3g). These results indicate that ZFHX3 repressed FTO expression through impairing the activity of the *FTO* promoter by directly binding to the FTO promoter.

**Fig. 4:**
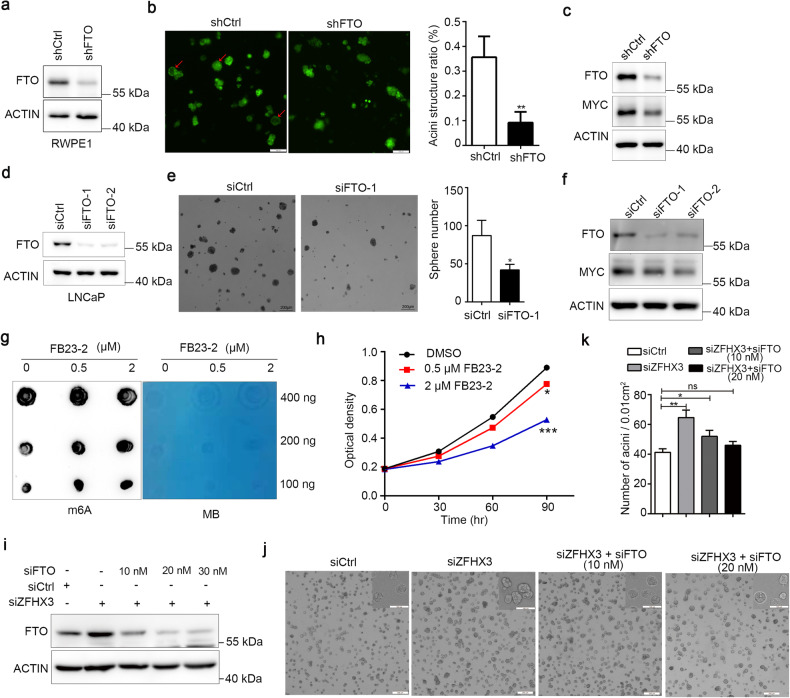
FTO inhibition impairs colony or acini formation in LNCaP and RWPE1 cells. **a**, **d** The knockdown efficiency of FTO shRNA with lentivirus constructs in RWPE1 or siRNAs against FTO in LNCaP was confirmed by western blot. **b** Acini formation was decreased in the FTO knockdown cell line. The arrows indicated the normal acini structure. *n* = 3. **c**, **f** Western blot showed that MYC expression was repressed when FTO was knocked down by shRNA or siRNAs, separately in RWPE1 and LNCaP. **e** Sphere formation was decreased after knocking down FTO (siFTO-1) in LNCaP. The average number of spheres with a diameter >75 µm/well was counted. *n* = 3. **g** We determined the m^6^A abundance in mRNA in RWPE1 cells upon FTO inhibitor FB23-2 treatment under various concentrations as indicated for 72 h via dot blot assay. **h** FB23-2 suppressed cell proliferation of RWPE1 detected by SRB assay. The cells treated with different concentrations of FB23-2 were collected at indicated time. *n* = 3.**i**–**k** Knockdown of ZFHX3 by siRNA was accompanied by three concentrations of siRNAs against FTO as indicated. The efficiency of ZFXH3 and FTO knockdown was tested by western blot and FTO knockdown eliminated the promoting effect of ZFHX3’s inhibition on acini formation in RWPE1 cells. *n* = 3. *P < 0.05; **P < 0.01; ***P < 0.001, ns not significant.

Furthermore, we investigated whether FTO was involved in ZFHX3’s tumor suppressor activity in PCa. We first explored the function of FTO in PCa and sought to estimate the altered cellular phenotypes in PCa cells depleted of FTO in vitro. FTO was efficiently knocked down in RWPE1 and LNCaP cells (Fig. 4a, d). RWPE1, as an immortalized prostate epithelial cell line, yielded structurally differentiated acini in 3D Matrigel culture [50]. FTO deficiency inhibited acinar morphogenesis of RWPE1 in 3D Matrigel (Fig. 4b). Meanwhile, the degree of sphere formation with a diameter >75 μm was decreased when silencing FTO in sphere formation assay within LNCaP cells (Fig. 4e). In the SRB assay, knockdown of FTO decreased cell proliferation in 22Rv1 cells (Fig. S3). Our previous study demonstrated that MYC downregulation was necessary for ZFHX3 to inhibit cell proliferation in PCa cells [47] and FTO inhibition also led to the downregulation of MYC signaling in AML cells [27]. Considering FTO expression was mediated by ZFHX3 and MYC as a target of FTO, we detected the expression of MYC in FTO knockdown cells and found FTO downregulation repressed MYC expression (Fig. 4c, f). In addition, we utilized a specific potent inhibitor of FB23-2 to further determine the role of FTO in PCa. Inhibition of FTO by FB23-2 increased the total m^6^A levels in a dose-dependent manner (Fig. 4g). We also detected the impact of FTO inhibitor on the growth of RWPE1 cells with SRB assay. FB23-2 treatment showed significant inhibitory effects on the growth of RWPE1 cells in a dose-dependent manner (Fig. 4h). Taken together, these results demonstrate the oncogenic role of FTO in PCa.

In our previous studies, we demonstrated the tumor suppressor activity of ZFHX3 in LNCaP/C4-2B PCa cells, but the role of ZFHX3 in RWPE1 cells had not been examined. In this regard, we knocked down ZFHX3 accompanied by different concentrations of FTO siRNA to reduce FTO expression in RWPE1 cells, and acini formations were conducted in Matrigel in vitro. The efficiency of ZFHX3 or FTO depletion was detected by western blot (Fig. 4i), and we found that acini formation was compensated when excess FTO expression was reduced, which was induced by ZFHX3 deficiency to a similar level compared to that of control cells (Fig. 4j, k). These results indicate that ZFHX3 exerts a suppressor role in PCa by regulating m^6^A modification through repressing FTO transcription and expression.

### FTO inhibition suppresses PCa cell proliferation

**Fig. 5:**
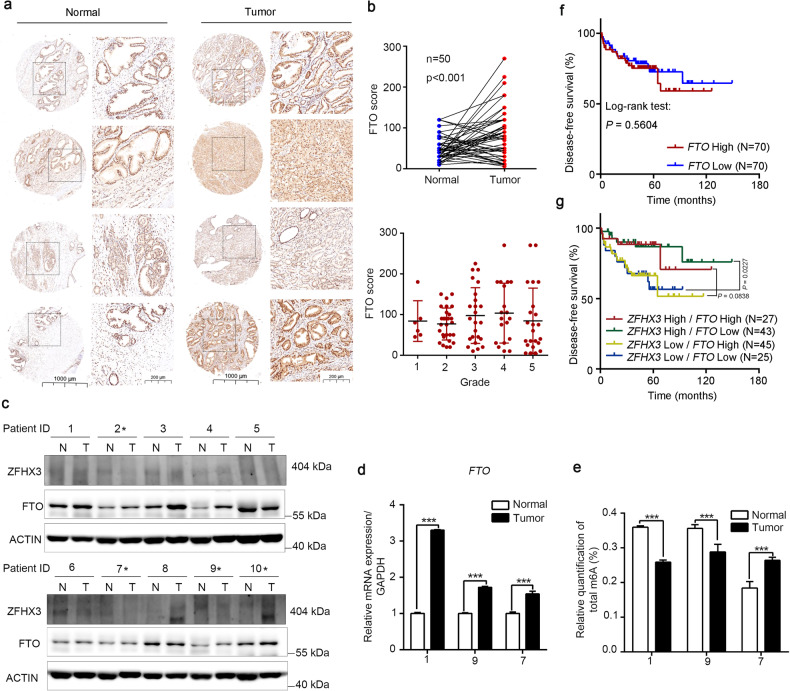
FTO expression is upregulated in PCa. **a** We performed IHC staining of FTO expression in PCa tumor tissue microarray (TMA), which contained 50 normal prostate specimens and 100 prostate tumor specimens. **b** The paired PCa tissues’ IHC scores for 50 patients showed that FTO was upregulated in tumors compared to adjacent normal tissues, but had no significant change in different grades. **c** Western blot assay was used to determine FTO expression in 10 patients and FTO expression was elevated in most tumor tissues compared to adjacent normal tissues. **d** FTO expression at the mRNA level was increased in tumor tissues of patient 1, patient 7, and patient 9. **e** Colorimetric assay was performed to detect the total m^6^A level in patient 1, patient 7, and patient 9. **f** High *FTO* expression was not correlated with better disease-free survival in PCa patients, as determined by the Kaplan–Meier analysis. **g** Kaplan–Meier analysis of disease-free survival of PCa patients with different statuses of *ZFHX3* and *FTO* expression was presented. The *n* of (**d**) and (**e**) is 3. ***P < 0.001.

To investigate the clinical implications of FTO in PCa, we applied immunohistochemistry (IHC) staining in a tissue microarray (TMA) including 50 normal prostate specimens and 100 prostate tumor specimens. The results revealed that FTO expression was enhanced in primary tumor tissues compared with the matched normal tissues but was not associated with prostate tumor grade (Fig. 5a, b). We also tested the expression of FTO and ZFHX3 in PCa patients and observed that FTO was aberrantly upregulated at the protein level in human PCa tissues compared to normal tissues from the same patient (Fig. 5c). This result may suggest that ZFHX3 expression had a negative relationship with FTO in some patients as indicated. FTO was elevated at the mRNA level in tumor tissues, but the m^6^A levels of a tumor tissue from patient 7 were not decreased compared to paired normal tissue (Fig. 5d, e). We investigated the potential relationship between *ZFHX3*/*FTO* expression and disease-free survival (DFS) in human PCa by analyzing the cohort of PCa patients for whom data are available for both gene expression and DFS. The data indicated that FTO expression was not correlated with patients’ DFS (Fig. 5f), but a Kaplan–Meier curve with log-rank analysis demonstrated that when ZFHX3 expression was higher, patients with lower FTO had significantly better DFS (Fig. 5g). These results suggested that patients with higher ZFHX3, regardless of FTO status, significantly correlated with better DFS.

### FTO mediates cell cycle progression by regulating related genes’ expression

**Fig. 6:**
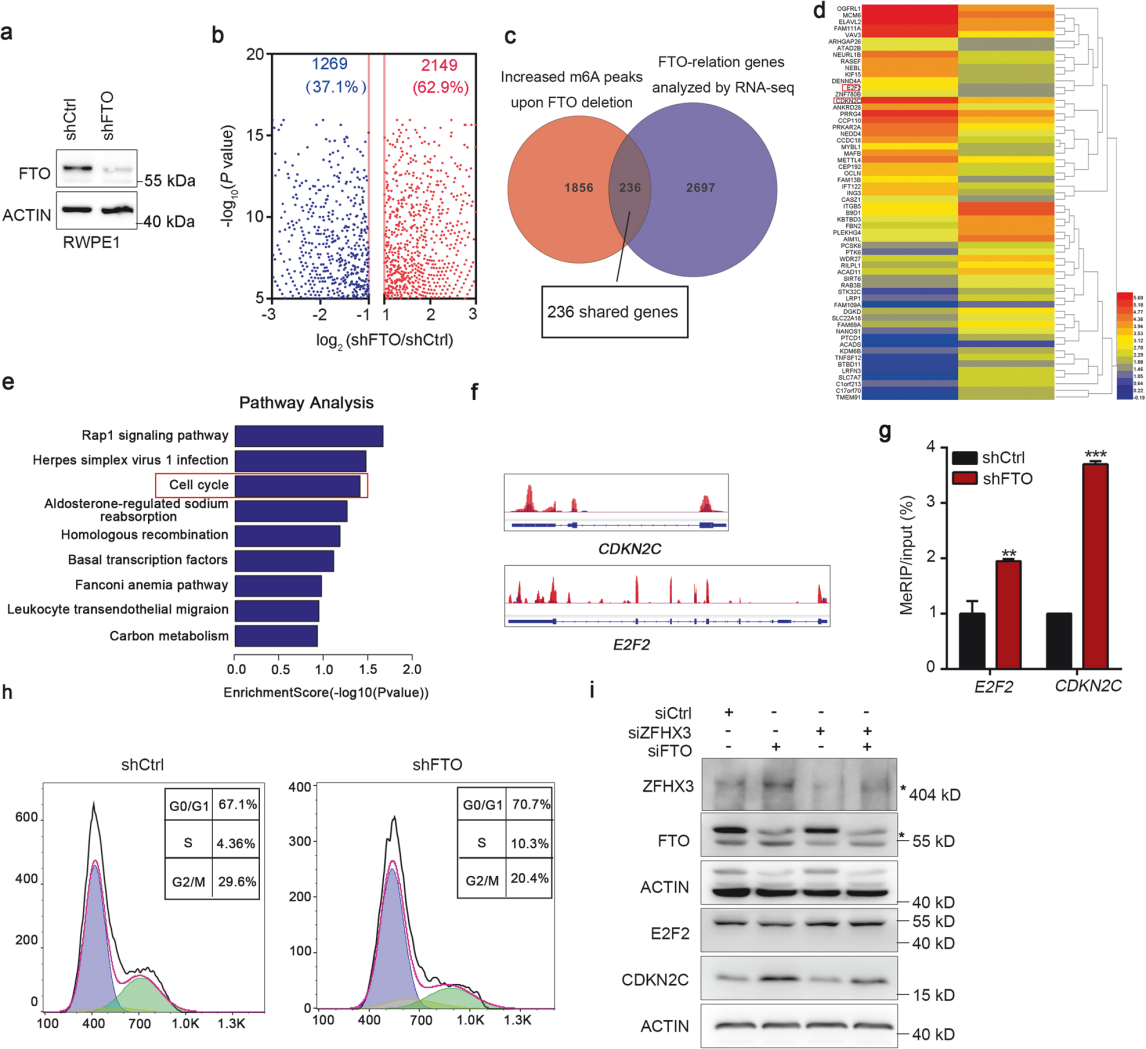
Identification of FTO targets via MeRIP-seq and RNA-seq. **a** Immunoblotting of FTO in FTO knockdown RWPE1 and control RWPE1 cells is presented. **b** The distribution of peaks is shown with a significant change in m^6^A level in FTO knockdown RWPE1 cells compared to control RWPE1 cells. **c** A Venn diagram shows the shared genes between increased m^6^A peaks upon FTO deletion and FTO-relation genes analyzed by RNA-seq. A total of 236 genes were observed. **d**, **e** Pathway analysis and KEGG analysis of the above 236 shared genes showed that the cell cycle was altered in FTO knockdown cells. **f** The mapped reads represent enriched RNA fragments by MeRIP-seq. RNA methylation profiles were loaded in IGV software and m^6^A modification peak alterations in *CDKN2C* and *E2F2* mRNA full length were visualized. **g** MeRIP-qRT-PCR was used to detect the m^6^A levels alterations of *CDKN2C* and *E2F2* after knocking down FTO in RWPE1 cells. **h** Cell cycle distribution of FTO silencing cells and control cells was analyzed by flow cytometry. **i** E2F2 and CDKN2C were detected by western blot in ZFHX3 knockdown cells or control cells where FTO was silenced with siRNA targeting FTO or siCtrl. **P < 0.01; ***P < 0.001, ns not significant.

To explore the underlying mechanisms of FTO in PCa development, we performed RNA sequencing (RNA-seq) and m^6^A mRNA immunoprecipitation sequencing (MeRIP-seq) in FTO knockdown RWPE1 cells, and the efficiency of FTO depletion was detected by western blot (Fig. 6a). We found 2149 hyper-methylated m^6^A peaks in FTO knockdown RWPE1 cells compared to control RWPE1 cells (Fig. 6b). We further investigated the altered genes, corresponding to the m6A-hypo genes induced by FTO deletion, in our RNA-seq data and found 236 genes shared between these two groups (Fig. 6c). These changed genes were displayed and visualized in the heatmap (Fig. 6d). Meanwhile, we discovered that the shared genes were enriched across the progression of the cell cycle as shown in the pathway analysis, which was consistent with the heatmap results (Fig. 6e). The obvious m^6^A peak increases of CDKN2C and full length E2F2 were induced by knockdown FTO and were visualized with IGV software (Fig. 6f); the m^6^A modification of CDKN2C and E2F2 was increased by quantification analysis (Fig. 6g). Furthermore, we performed an FACS assay to address whether FTO knockdown influenced the cell cycle and found an increase in the number of cells in the S phase and a decrease in the number of cells in the G2/M phase (Fig. 6h). More importantly, we found that FTO knockdown induced an increase of CDKN2C and impaired E2F2 expression, but the effect was partly inhibited by ZFHX3 deficiency (Fig. 6i). These results indicate that the role of FTO on PCa cell proliferation through regulating CDKN2C and E2F2 depends on ZFHX3.

### FTO represses ZFHX3 through an m6A-mediated mechanism

**Fig. 7:**
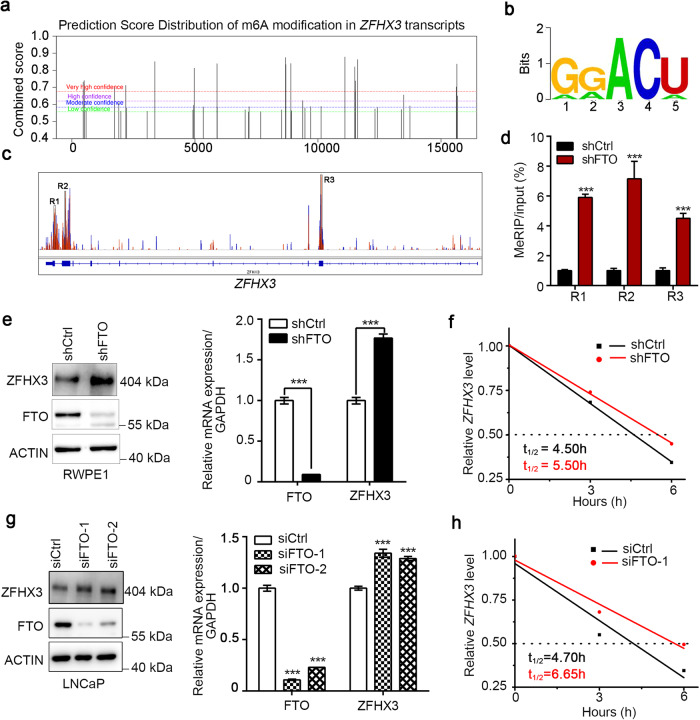
FTO mediates the mRNA stability of ZFHX3 in an m6A-dependent way. **a**, **b** The potential m^6^A sites of *ZFHX3* and the consensus motifs modified by m^6^A predicted by SRAMP are shown. **c** The m^6^A peaks in the black rectangles (R1, R2, and R3) visualized by IGV are those that co-localized with predicted sites of *ZFHX3*. **d** The m^6^A levels of fragments in *ZFHX3* (R1, R2, and R3) were elevated by MeRIP-qRT-PCR after knocking down FTO in RWPE1 cells. *n* = 3. **e** Western blot and RT-qPCR assays showed that ZFHX3 expression at the protein level or mRNA level was elevated after depletion of FTO (shFTO) in RWPE1 cells. *n* = 3. **f** The mRNA half-life (*t*_1/2_) was increased in RWPE1 cells with depleted expression of FTO (shFTO). **g** ZFHX3 protein expression or mRNA expression was upregulated after knockdown of FTO (siFTO-1 and siFTO-2) in LNCaP cells. *n* = 3. **h** The mRNA half-life (*t*_1/2_) was increased in LNCaP cells with depleted expression of FTO (siFTO-1). ***P < 0.001.

To further investigate the relationship between ZFHX3 and FTO, we used the online m^6^A sites prediction tool SRAMP (http://www.cuilab.cn/sramp) [51] and identified the conservative predictions that may be modified by m^6^A in ZFHX3 transcripts (Fig. 7a, b). We analyzed the MeRIP-seq of FTO knockdown in RWPE1 cells and found that three m^6^A peaks (R1, R2, and R3) were well matched and overlapped with conservative consequences in ZFHX3 (Fig. 7c). To test whether this regulation was m6A-dependent, MeRIP-RT-qPCR was performed, and the results indicate that FTO knockdown significantly enhanced the m6A-modified mRNA enrichment of ZFHX3 more than the shCtrl group in RWPE1 cells (Fig. 7d). To elucidate the role of FTO on ZFHX3 expression, we validated that FTO knockdown upregulated the protein level and mRNA level of ZFHX3 by western blot and qRT-PCR in RWPE1 and LNCaP cells (Fig. 7e, g). To further analyze the effect of the m^6^A level increase on the stability of *ZFHX3* transcription, we conducted RNA stability assays and found that the knockdown of FTO prolonged the half-life of ZFHX3 transcripts in RWPE1 and LNCaP cells (Fig. 7f, h). Thus, FTO-induced increases in ZFHX3 expression are, at least in part, due to the enhanced stability of ZFHX3 transcripts following the FTO-mediated increase *ZFHX3’* m^6^A levels.

## Discussion

ZFHX3 has been established as a tumor suppressor in PCa, and our previous studies demonstrate that ZFHX3 represses PCa cell proliferation through inhibiting MYC expression. However, more supportive evidence for the role of ZFHX3 in PCa is still needed. In this study, we report that ZFHX3 modulates m^6^A modification through regulating FTO expression in PCa. This study could help us to better understand the functions of ZFHX3 and m^6^A modification in PCa development and provide a candidate biomarker for PCa therapy.

We found four m^6^A-related genes which, as predicted by the analysis of ChIP-seq, may be downstream targets of ZFHX3. Considering the total m^6^A reduction and translocation from the nucleus to the cytoplasm when ZFHX3 is knocked down in RWPE1 or LNCaP cells, we focused on the role of main demethylase FTO in ZFHX3-mediated m^6^A modification. However, we cannot ignore the roles of m^6^A methylases and have much work to do in evaluating the functions of m^6^A methylases in ZFHX3-mediated m^6^A modification.

We verified that ZFHX3 deficiency increased the mRNA and protein levels of FTO through enhancing the activity of *FTO* promoters, and we confirmed the critical FTO promoter region regulated by ZFHX3. More importantly, FTO knockdown by siRNA at moderate concentration could rescue the acini formation increase that is induced by ZFHX3 inhibition. Furthermore, our results also suggest that ZFHX3 was negatively associated with FTO in PCa patients, but we need more evidence on this conclusion. Taken together, this evidence indicates that FTO may be involved in ZFHX3-mediated tumor progression in PCa.

FTO is the key component of demethylases in m^6^A modification and has been reported to be essential for cancer progression [27, 52–55], including in hepatocellular carcinoma, AML, glioblastoma, and lung cancer. Nevertheless, there is little evidence about the role of FTO in PCa. Two recent studies stated that FTO inhibited the invasion and migration of PCa [35, 56]. In this study, we demonstrated that FTO expression was high in PCa and promoted cell proliferation in LNCaP and RWPE1 cells, which may be contrary to previous reported studies. To investigate this contradiction on the role of FTO in PCa, we detected the function of FTO in PC-3 and found that FTO knockdown provoked cell proliferation and promoted sphere formation (Fig. S4). These results suggest that FTO may play different roles in androgen receptor (AR)-positive cells and AR-negative cells. In addition, whether FTO plays a consistent role in the whole progression of PCa from initiation of PCa to metastatic castration-resistant prostate cancer requires further study. These questions are needed much more studies on the role of FTO in PCa. However, ZFHX3 performs a tumor suppressor role in both AR-positive cells (Fig. 2) and AR-negative cells (Fig. S5). This conflict may be related to ZFHX3 being a transcription factor which may regulate other genes’ expression in AR-negative cells.

Mechanistically, ZFHX3 exerts its tumor suppressor role partly through inhibiting the enzymatic activity of FTO. FTO knockdown enhanced the accumulation of m^6^A on some transcripts, including CDKN2C and E2F2, thus inducing cell cycle arrest. However, the regulation of FTO knockdown on CDKN2C and E2F2 is repressed by ZFHX3 silencing, which indicates that ZFHX3 impaired FTO’s effect on PCa growth. In addition, the expression of ZFHX3 was also regulated by FTO-mediated m^6^A modification, and FTO deletion increased ZFHX3 expression. These findings suggest that there is a feedback loop between ZFHX3 and FTO expression, and the balance between ZFHX3 and FTO influences PCa cell proliferation.

In summary, we report a crosstalk between ZFHX3 and FTO-mediated m^6^A modification in PCa cells. ZFHX3 elevated the total m^6^A levels through repressing FTO expression, and in return, the decrease of FTO enhanced the m^6^A modification in ZFHX3 transcripts and provoked ZFHX3 stabilization at the mRNA level, resulting in an increase of ZFHX3 expression. We uncovered a novel mechanism underlying ZFHX3’s tumor suppressor role in PCa. In addition, we stated the oncogenic role of FTO in PCa and uncovered that two key regulators, E2F2 and CDKN2C, identified as targets of FTO under the control of ZFHX3 play an important role in repressing the cell cycle in vitro. Further studies will focus on the other enzymes’ dysregulation and explore the combined actions of specialized enzymes on tumors, aiming to find the potential therapeutic benefit of targeting these critical enzymes.

**Fig. 8:**
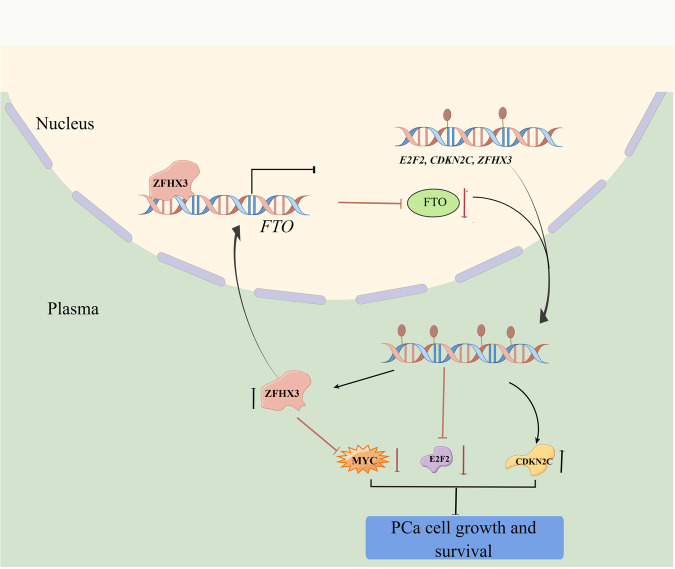
Proposed model of the feedback loop between ZFHX3 and FTO-mediated m^6^A modification in PCa cells. ZFHX3 suppressed the transcription of *FTO*, which increased the m^6^A modification of target genes, including *ZFHX3*, *E2F2*, and *CDKN2C*. Meanwhile, ZFHX3 was regulated by FTO through m^6^A modification.

The data we report advances our understanding of how ZFHX3 inhibits PCa in an FTO-mediated m^6^A modification manner, and the negative feedback loop between ZFHX3 and FTO suggests that the function of FTO in PCa partially depends on the status of ZFHX3, as depicted in Fig. 8.

## Material and methods

### Tissue microarray and tissue samples collection

A TMA (HProA150CS01) was purchased from Outdo Biotech Company (Shanghai, China) containing 50 paired PCa tumor tissues and normal tissues adjacent to cancer and 50 individual PCa tumor tissues. Samples with PCa had been graded according to the international staging system criteria. This TMA was used to evaluate FTO expression in the tumor and normal tissues.

PCa tissues and matched normal tissues were collected from ten PCa patients who underwent prostate surgery at the Department of Urology of Shandong Provincial Hospital of Shandong First Medical University. We performed the study in accordance with the Helsinki Declaration and the research was also authorized by the Ethics Committee of Shandong Provincial Hospital of Shandong First Medical University (NSFC: No. 2020-1322). We had obtained all the patients’ informed consent. Tissue samples collected during surgery were all diagnosed by histopathology and then stored in liquid nitrogen before use.

### Immunohistochemical staining

FTO expression in TMA was detected by IHC. Tissue sections were first deparaffinized and rehydrated in graded ethanol following standard procedure. Antigen retrieval was carried out by heating the sections with sodium citrate buffer and the sections were blocked by hydrogen peroxide to eliminate endogenous peroxidase. Next, sections were successively incubated with primary anti-FTO antibody (1:500, ab124892, Abcam) and with the goat anti-rabbit IgG (1:1000, SA00004-2, Proteintech). Sections were stained with the DAB-chromogen.

Two pathologists, without access to patient data, separately evaluated the stained results independently by single-blinding to the clinicopathological data. We graded the staining intensity according to previous reports [57, 58]. The staining strength from strong to negative was scored ranging from 3 to 0. The percentage of stained area was assessed according to density ranging from 0 to 100%. Finally, the score was calculated by multiplying two scores.

### Cell culture, transfection, and infection

Human immortalized prostate epithelial cell line RWPE1 and other human PCa cell lines were purchased from KeyGEN Biotech (Jiangsu, China). RWPE1 cells were cultured in Keratinocyte-SFM medium (2101, ScienCell, CA, USA) with KGS. LNCaP cells, PC-3 cells, and 22Rv1 cells were cultured in a complete RPMI-1640 medium (Keygen Biotech, Jiangsu, China). DU 145 cells were cultured in a complete RPMI-1640 medium (Keygen Biotech, Jiangsu, China). The identities of cell lines were confirmed by short tandem repeat analysis.

We synthesized RNAis targeting human ZFHX3 and FTO from RiboBio (Guangzhou, China). The siRNA sequence for silencing ZFHX3 (siZFHX3) was 5′-AGAAUAUCCUGCUAGUACA-3′, which had been validated in our study [59]. The siRNA sequences against human FTO were 5′-GCUGAAAUAUCCUAAACUA-3′ and 5′-GAACUCAGAACACCCAAUA-3′. Cells were transfected by using the Lipofectamine RNAiMAX reagent (Invitrogen).

We purchased lentivirus-based GV248 shRNA vector from Genechem (Shanghai, China) and target sequence for FTO was GCAGCATACAACGTAACTTTG. ShRNAs targeting ZFHX3 in pLKO.1-puro plasmid and vector control plasmid were gifts from Dr. Jin-Tang Dong. Sequences of shRNAs against ZFHX3 are listed below: #1: CCGGGCCAGGAAGAATTATGAGAATCTCGAGATTCTCATAATTCTTCCTGGCTTTTT and #2: CCGGCCCTTTAGTTTCCACAGCTAACTCGAGTTAGCTGTGGAAACTAAAGGGTTTTT, which have been previously described [41]. RWPE1 cells were infected with lentivirus targeting *FTO* or vector, and then screened out the stable FTO-depleted cells under 1 μg/mL puromycin condition. Finally, the stable cell lines were cultured with 0.4 μg/mL puromycin.

### Establishment of cell lines

To generate 22Rv1 stable cell lines that ectopically express ZFHX3. 22Rv1 cells were transfected with pcDNA3.0-Flag and Flag-tagged ZFHX3 plasmids (gifts from Dr. Jin-Tang Dong) and then selected with 400 μg/mL G418 for 10 days. Stable clones were isolated and identified by western blotting analysis.

### Animal experiments

The animal studies were approved by the Animal Ethics Committee of Shandong Provincial Hospital (NSFC: No. 2020-1111). 2 × 10^6^ PCa cells (22Rv1 cells transfected with Flag-vector and Flag-ZFHX3) in 100 μL PBS/Matrigel (1:1) were injected into the flanks of the 4-week-old male NOD-scid mice. Tumor volumes (*V* = 0.5 × length × width^2^) were measured every 7 days. After 4 weeks, the mice were sacrificed, and the tumors were isolated and weighed. Western blot was used to detect the characteristics of xenograft tumors.

### Western blot assay

The procedures were performed according to our previous study [47]. The primary antibodies used in western blot were as follows: anti-ACTIN (20536-1-AP, Proteintech), anti-ZFHX3 (PD010, MBL), anti-FTO (27226-1-AP, Proteintech), anti-MYC (TA150121, OriGene), anti-E2F2 (ab138515, Abcam), and anti-CDKN2C (ab192239, Abcam).

### Immunofluorescence staining

Cells were washed with PBS (phosphate-buffered saline) and then fixed with 4% paraformaldehyde, permeabilized with 0.5% Triton X-100. Cells were blocked with 5% bovine serum albumin in PBS at room temperature and then incubated with primary antibodies (1:200 dilution, 56593S, CST) overnight at 4 °C. After washing, cells were incubated with secondary antibodies conjugated with Alexa Fluor™ 594 (1:1000, A-11012, Thermo Fisher) at room temperature for 2 h. We used 4′,6-diamidino-2-phenylindole (DAPI) (ab104139, Abcam) to stain nuclei for 10 min. The images were taken with fluorescence microscope (Leica, Germany).

### Acinar morphogenesis and sphere formation in Matrigel

The sphere formation assay protocol for LNCaP cells has been described in our previous study. RWPE1 cells were detached from flasks and suspended the cells at 2500 cells/100 μL. Fourty microliters of Standard BD Matrigel Matrix (BD Biosciences, Sparks, MD) was added to each well of 8-well glass chamber slides. After Matrigel was solidified for 1 h at 37 °C, 400 μL of cell suspension in complete K-SFM medium was seeded into each well. The images were taken at 7 days with microscope (Leica, Germany).

### RNA extraction and quantitative RT-PCR

Total RNAs were isolated using the RNA extraction Kit (SparkJade, Shandong, China) according to the manufacturer’s instructions. Two hundred nanograms RNA as templates were reverse-transcribed into DNA in a total reaction volume of 20 μL with RT kit (Vazyme, Jiangsu, China). qRT-PCR analysis was performed with 1 μL cDNA using SYBR green (Vazyme) in an Roche 480 real-time PCR instrument (Applied Biosystems, Foster City, CA). The primers for RT-PCR were listed in Supplementary Table 1.

### RNA m^6^A dot blot assay and quantification

Total RNA was isolated from different cells as described. The m^6^A dot blot assay was performed following a published protocol [12]. Briefly, the RNA samples in certain volume were loaded to the PVDF membrane (IPVH0010, Milipore) and Ultraviolet crosslinker was used to crosslink RNA and membrane. Then the membrane was blocked and incubated with anti-m^6^A antibody (1:1000 dilution, ab208577, Abcam) overnight at 4 °C. Then the membrane was incubated with secondary antibody at room temperature, and the membrane was detected with ECL Detection Reagent (PK10002, Proteintech). The signal of each dot was tested by Minichem^TM^ Chemiluminescence Imaging System (Sagecreation, Beijing, China) in all experiments.

The m^6^A RNA methylation quantification kit (p-9005, EpigenTek) was used to detect the m^6^A content. Two hundred nanogram RNAs were added to the wells. We conducted RNA m^6^A quantification assay following the manufacturer’s protocol. The m^6^A content was quantified by measuring the absorbance at 450 nm.

### RNA stability assays

The mRNA stability was used to test the degrading half of the existing mRNA molecules. FTO-deleted cells and control cells were seeded in 6-well plates. Then actinomycin D was added to cells at 5 μg/mL concentration and cells were collected at 6, 3, and 0 h. Total RNA was isolated and RT-PCR was conducted to test the relative quantity of target mRNA. The half-life and turnover rate of ZFHX3 mRNA was evaluated according to a previously published paper [6].

### m^6^A MeRIP sequencing, RNA sequencing, and data analysis

MeRIP-seq and RNA-seq were performed by Cloudseq Biotech Inc. (Shanghai, China). Briefly, MeRIP-seq was conducted using the GenSeq^TM^ m^6^A RNA IP kit (GenSeq lnc., China). Both the input without immunoprecipitation and the IP samples were used for MeRIP-seq library generation using NEBNext® Ultra II Directional RNA Library Prep kit (New England Biolabs). For RNA-seq, library was constructed using the TruSeq Stranded Total RNA Library Prep Kit (Illumina, San Diego, CA). The quality of both libraries was evaluated using the BioAnalyzer 2100 system (Agilent Technologies, Inc.). Library sequencing was performed on an Illumina Hiseq instrument with 150 bp paired-end reads. Datasets were deposited to the NCBI Gene Expression Omnibus database (GSE244356).

### MeRIP-RT-qPCR

The MeRIP assay was performed according to the protocol of RNA Immunoprecipitation Kit (GS-ET-006, Cloudseq, Shanghai, China). Cells cultured in 15 cm dishes were collected with PBS, then scraped with RIP lysis buffer which containing protease inhibitor and RNase inhibitors. Both m^6^A antibody and mouse IgG were incubated with magnetic beads for 1 h at room temperature and then incubated with cell lysate at 1 h at room temperature. After two washes, the beads were eluted and the input and immunoprecipitated RNAs were recovered, extracted, and subjected to qRT-PCR analysis. The primers for qRT-PCR were listed in Supplementary Table 1.

### Luciferase reporter gene assays

Mammalian expression plasmids for promoter plasmid for *FTO*-wild type and *FTO*-mutant type were constructed using a PCR and the primers for plasmids construction were listed in Supplementary Table 2. The vector GV238 (MCS-firefly-luciferase) was purchased from Genechem (Shanghai, China). LNCaP cells were transfected with Luc-*FTO*-Wt or Luc-*FTO*-Mut and pRL-TK as an internal control for 24 h. The luciferase activities of the reporters were determined by using the dual-luciferase reporter gene assay kit (Promega, Madison, WI, USA). Luciferase activities were normalized to Renilla luciferase activity. Experiments were performed in triplicate.

### Chromatin immunoprecipitation (ChIP) assay

293T cells were transfected with control-vector or Flag-ZFHX3 plasmid. A ChIP assay was conducted according to the manufacturer’s instruction of ChIP-IT Express Enzymatic Chromatin IP Kit (53009, Active Motif, MA, USA). 293T cells transfected for 48 h were cross-linked with 1% formaldehyde. The samples were quenched with glycine, and the cross-linked material was sonicated into chromatin fragments. Chromatin extracts were separately incubated with ZFHX3 antibody and normal rabbit IgG as a negative control. qRT-PCR was performed for detecting the quantity of DNA binding to ZFHX3. The primers were listed in Supplementary Table 3.

### Patient survival and statistical analysis

The survival analysis was performed by using a published cohorts of prostate patients which included 140 PCa samples that had both mRNA expression data and DFS status. Survival estimates were performed by using the Kaplan–Meier curves, and survival curves were compared using a log-rank test.

All statistics for comparing the significant differences of two groups used unpaired Student’s *t*-test. Analysis of variance (ANOVA) was used to identify mean difference between groups. All statistical analyses were conducted using GraphPad Prism 6.

### Supplementary information

Supplementary materials

## Data Availability

All data generated or analyzed during this study are included in this published article.
