# Supplementary material for: ZFHX3 acts as a tumor suppressor in prostate cancer by targeting FTO-mediated m6A demethylation
Source: Cell Death Discov. 2024 Jun 13;10:284. doi: 10.1038/s41420-024-02060-w (PMC11176170; doi:10.1038/s41420-024-02060-w)
Supplement: Supplementary file 1 — Supplementary materials [file 41420_2024_2060_MOESM1_ESM.pdf]

Supplementary Figure 1

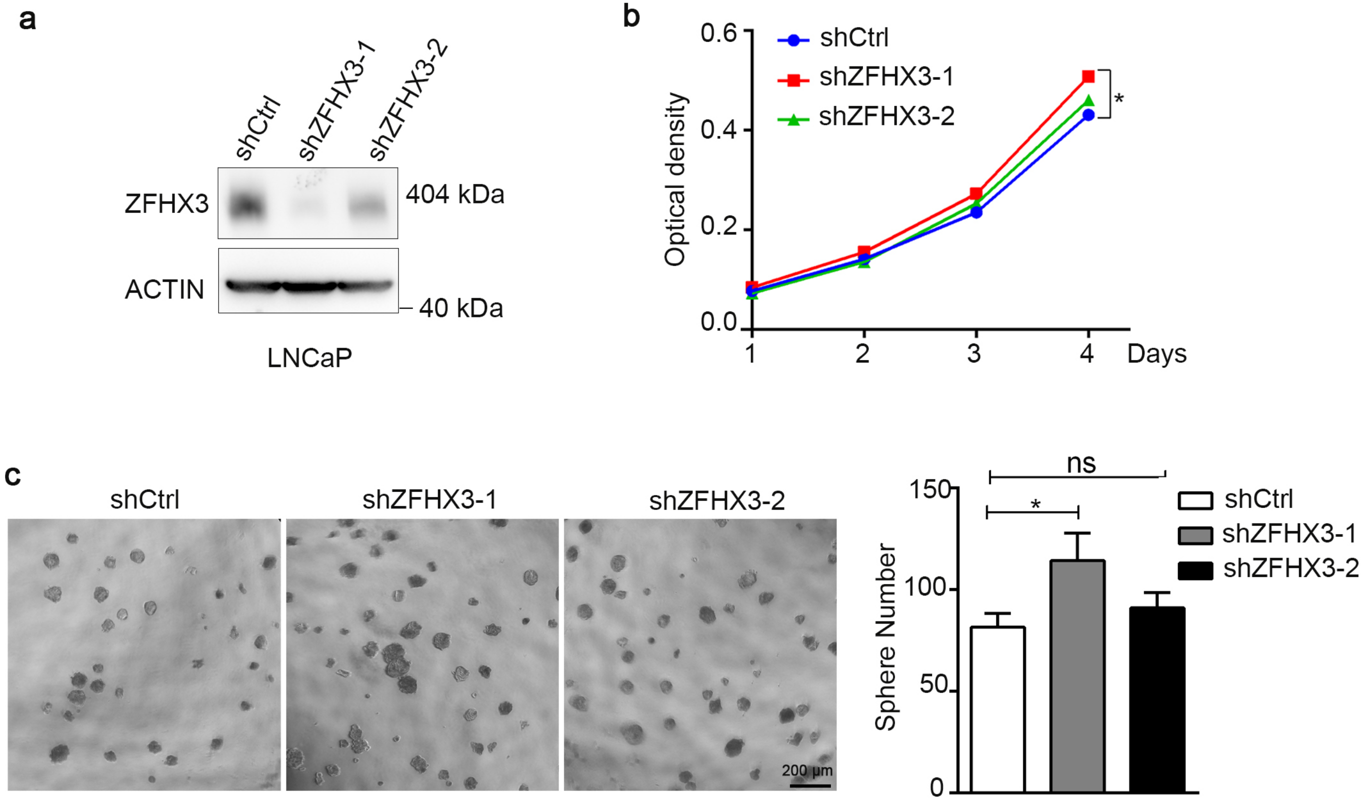

Supplementary Figure 2

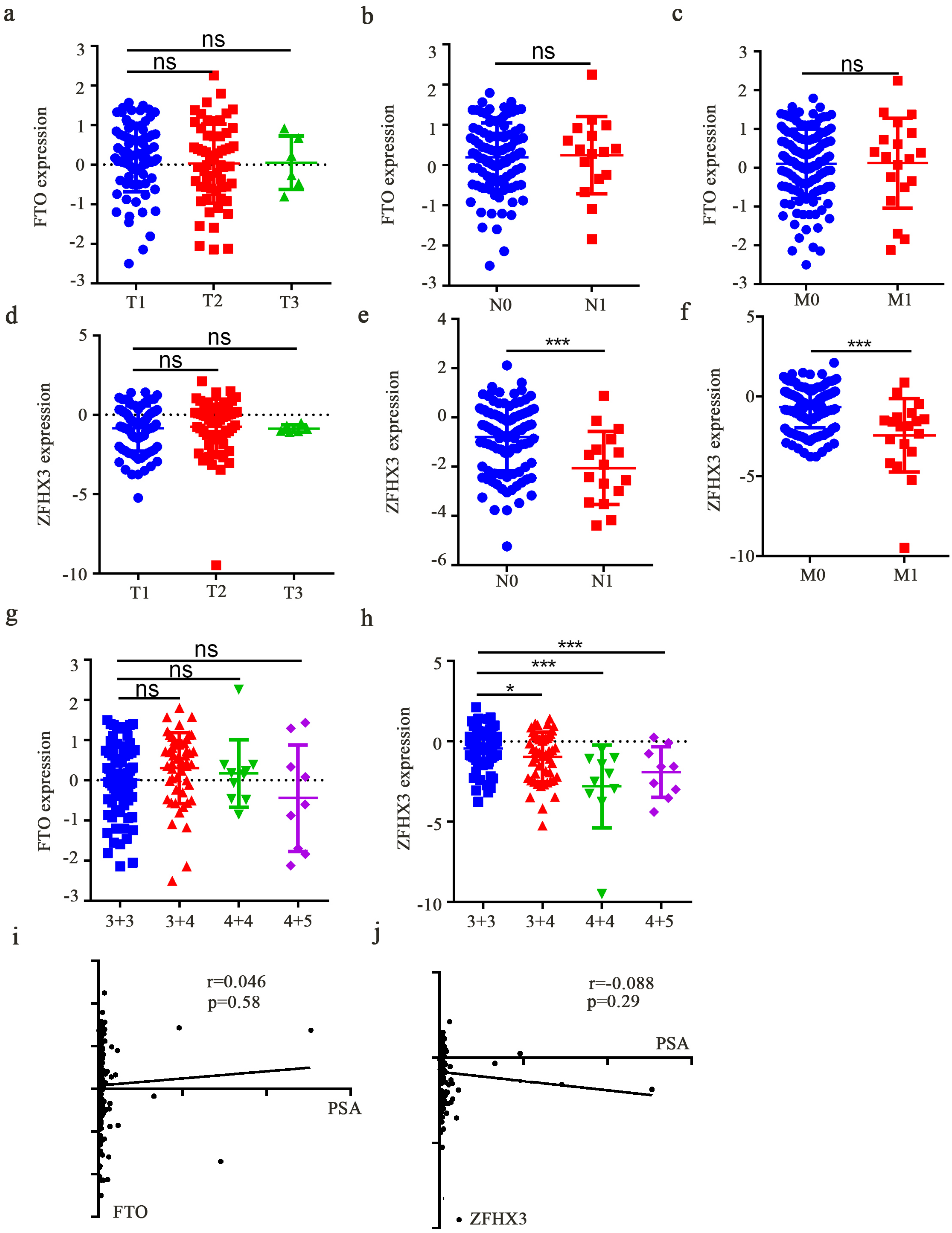

Supplementary Figure 3

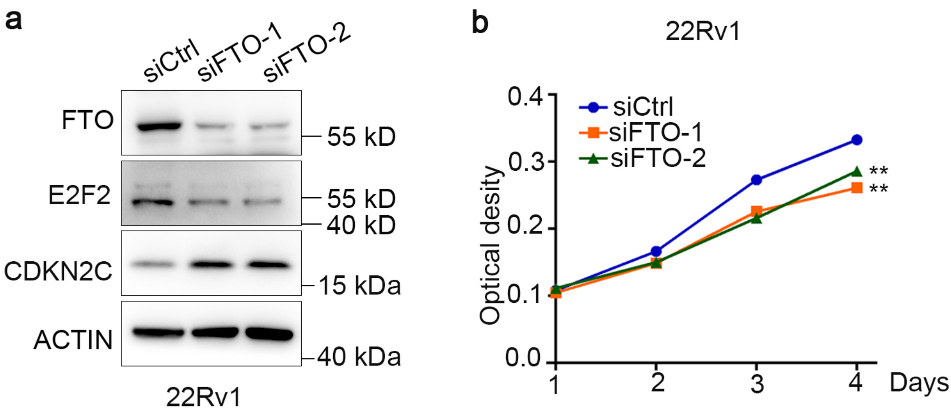

Supplementary Figure 4

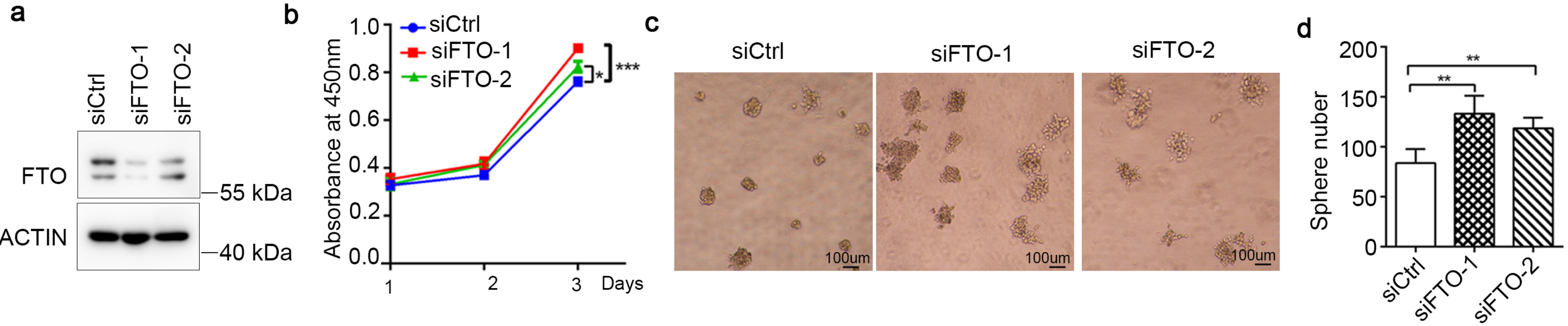

Supplementary Figure 5

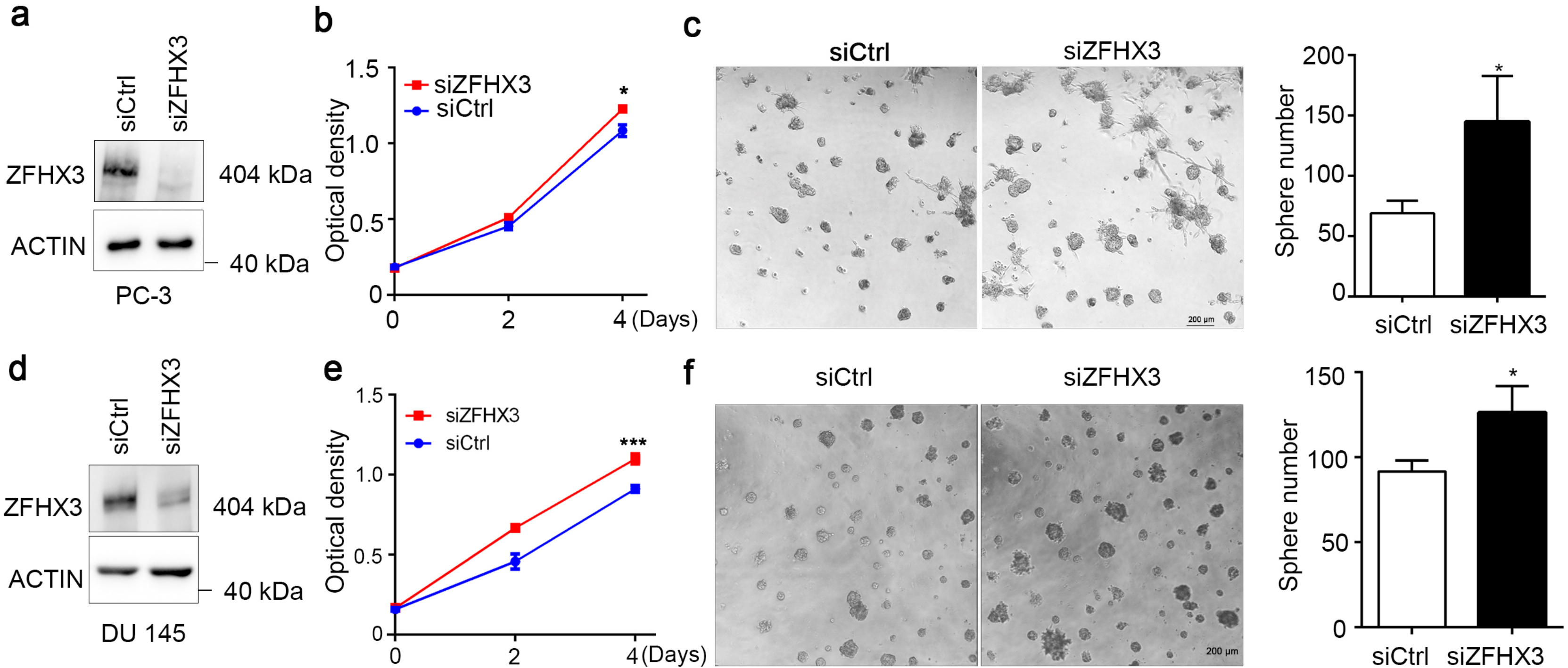

Supplementary figures

Supplementary Figure S1. Knockdown of ZFHX3 promoted cell proliferation in LNCaP cells. (a) The efficiency of ZFHX3 knockdown by two separate shRNAs was detected by western blot and shZFHX3-1 showed the most efficiency. (b) ZFHX3 knockdown promoted cell proliferation, as measured by SRB assay. **, $P < 0.01$. (c) Knockdown of ZFHX3 increased sphere formation in Matrigel. The data were presented as the average number of spheres with a diameter > 75 µm per well. n=3.

Supplementary Figure S2. **ZFHX3 and FTO expression in prostate cancer patients.** (a-c) Analysis of the Prostate dataset GSE21032. FTO mRNA expression (mRNA expression z-scores relative to all samples) was not correlated with tumor-node-metastasis (TNM) stage. (d-f) ZFHX3 expression was significantly decreased in prostate cancer patients with node and metastasis stage, but was not correlated with tumor stage. (g, h) The expression of FTO was not correlated with Gleason Score (g), and ZFHX3 expression was correlated with Gleason Score (h). (i,j) Neither ZFHX3 or FTO was correlated with PSA level in PCa patients containing mRNA expression and PSA level at diagnosis.

Supplementary Figure S3. Knockdown of FTO decreased cell proliferation in LNCaP cells. (a) The efficiency of FTO knockdown by two separate siRNAs was detected by western blot. CDKN2C expression was increased and E2F2 expression was decreased after knocking down FTO in 22Rv1 cells. (b) FTO knockdown inhibited cell proliferation via SRB assay.

Supplementary Figure S4. FTO knockdown promoted cell proliferation and sphere formation in PC-3 cells. (a) The efficiency of FTO knockdown by siRNAs against FTO was detected

by western blot. (b) FTO knockdown provoked cell proliferation by SRB assay. The cells were transfected with siRNAs and collected as indicated time. (c-d) FTO deficiency promoted sphere formation. Cells transfected with siRNAs were grown for 10 days in Matrigel. The bright filed images were shown (c) and the number of spheres with a diameter > 75 μm was counted by Image J (d). n=3. *, P < 0.05; **, P < 0.01; ***, P < 0.001.

Supplementary Figure S5. Knockdown of ZFHX3 promoted cell proliferation and sphere formation in PC-3 and DU 145. (a, d) The efficiency of ZFHX3 knockdown by siRNA was detected by western blot in PC-3 and DU 145. (b, e) ZFHX3 knockdown promoted cell proliferation by SRB assay. The cells were collected as indicated time. (c, f) ZFHX3 knockdown increased sphere formation. Cells transfected with siRNAs were grown for 10-14 days in Matrigel. The number of spheres with a diameter > 75 μm was counted by Image J. n=3. *, P < 0.05; **, P < 0.01; ***, P < 0.001.

Supplementary Table 1: Primer sequences for qRT-PCR

Names	Sequence
E2F2-FOR	GCAGATATATCTCAAGAGCACCC
E2F2-REV	GCTCCATGATGCTAGGGTCGGTG
CDKN2C-FOR	GCAGCAAAGGGGCACAGG
CDKN2C-REV	GCCAAGTTCTGAGTAGGGAGAG
ZFHX3-FOR	TGTTCCAGATCGAGATGGGAAT
ZFHX3-REV	CTTTCCCAGATCCTCTGAGGTTT
FTO-FOR	AGAATGTCTGTGACGATGTGG
FTO-REV	GCACTTTCTGTATCGATTGCC
GAPDH-FOR	GGTGGTCTCCTCTGACTTCAACA
GAPDH-REV	GTTGCTGTAGCCAAATTCGTTGT
RIP-E2F2-FOR	GCTGGTGGGGCTGGGAAGTTTCCTG
RIP-E2F2-REV	CTGACCTCAAGTGATCCACCACC
RIP-CDKN2C-FOR	GCGAGAGGTGGCAAAGTGGCAC
RIP-CDKN2C-REV	GCTGTCCTTTCCTTCCTTTCCC

Supplementary Table 2: Primer sequences used in gene clone

Names	sequences
FTO-P1	TTTCTCTATCGATAGGTACCGGAAGTACTCCTATAGAAAAGGTC
FTO-P2	CTTAGATCGCAGATCTCGAGACAGGAGAATTTCCCAGGTCCGAC

Supplementary Table 3: Primer sequences used in ChIP

Primer	Forward	Reverse
P1	GGCCATGGATGATGCTAAATATC	GCATAGTGAGCAAAGAAGGATAAC
P2	GCCTCTATCTGAAGTTATCAG	CCTGTGAGTATAAATCATCGCAGG
P3	CGTTGCTATAGCGCCGACAGC	CCACGGGATTTAGCACAGGAG

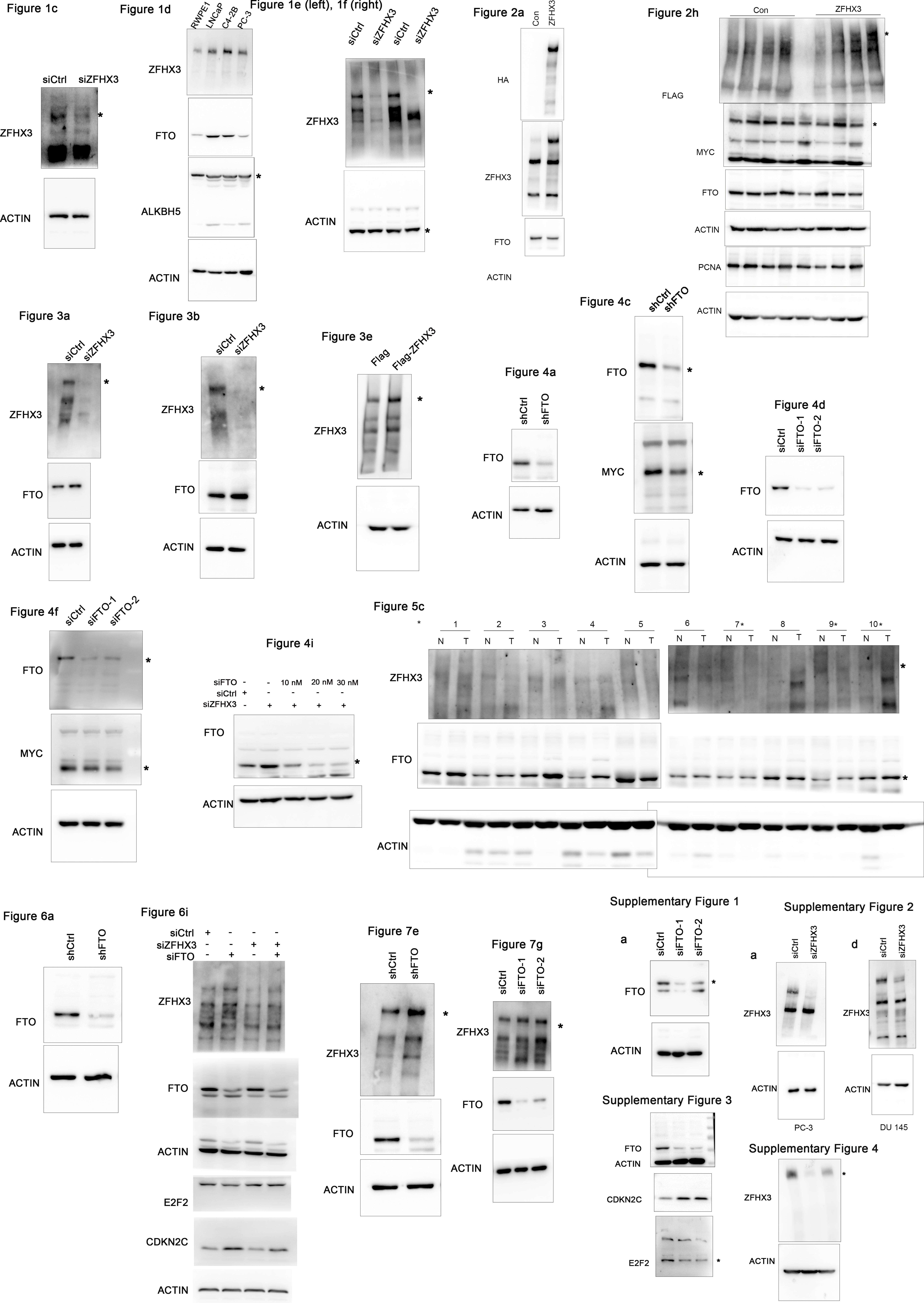
Figure 1c
siCtrl siZFHX3
ZFHX3
*
ACTIN
Figure 1d
RWPE1 LNCaP C4-2B PC-3
ZFHX3
FTO
ALKBH5
*
ACTIN
Figure 1e (left), 1f (right)
siCtrl siZFHX3 siCtrl siZFHX3
ZFHX3
*
ACTIN
*
Figure 2a
Con ZFHX3
HA
ZFHX3
FTO
ACTIN
Figure 2h
Con
ZFHX3
FLAG
*
MYC
*
FTO
ACTIN
PCNA
ACTIN
Figure 3a
siCtrl siZFHX3
ZFHX3
*
FTO
ACTIN
Figure 3b
siCtrl siZFHX3
ZFHX3
*
FTO
ACTIN
Figure 3e
Flag Flag-ZFHX3
ZFHX3
*
ACTIN
Figure 4a
shCtrl shFTO
FTO
ACTIN
Figure 4c
shCtrl shFTO
FTO
*
MYC
*
ACTIN
Figure 4d
siCtrl siFTO-1 siFTO-2
FTO
ACTIN
Figure 4f
siCtrl siFTO-1 siFTO-2
FTO
*
MYC
*
ACTIN
Figure 4i
siFTO - - 10 nM 20 nM 30 nM
siCtrl + - - - -
siZFHX3 - + + + +
FTO
*
ACTIN
Figure 5c
*
1 2 3 4 5 6 7* 8 9* 10*
N T N T N T N T N T N T N T N T N T N T
ZFHX3
*
FTO
*
ACTIN
Figure 6a
shCtrl shFTO
FTO
ACTIN
Figure 6i
siCtrl + - - -
siZFHX3 - - + +
siFTO - + - +
ZFHX3
FTO
ACTIN
E2F2
CDKN2C
ACTIN
Figure 7e
shCtrl shFTO
ZFHX3
*
FTO
ACTIN
Figure 7g
siCtrl siFTO-1 siFTO-2
ZFHX3
*
FTO
ACTIN
Supplementary Figure 1
a
siCtrl siFTO-1 siFTO-2
FTO
*
ACTIN
Supplementary Figure 3
FTO
ACTIN
CDKN2C
E2F2
*
Supplementary Figure 2
a
siCtrl siZFHX3
ZFHX3
ACTIN
PC-3
d
siCtrl siZFHX3
ZFHX3
ACTIN
DU 145
Supplementary Figure 4
ZFHX3
*
ACTIN

Author contribution statement

Q. H. and S. H. conceived the project; Q. H., K. Y., and S. H. participated in determining the experimental design and analyzed all data; Q. H., S. Z., R. S., J, Y., and Y. W. performed experiment; Q.H. and S. H. prepared the manuscript. All authors participated in contributing to text and the content of the manuscript, including revisions and edits. All authors approve of the content of the manuscript and agree to be held accountable for the work.

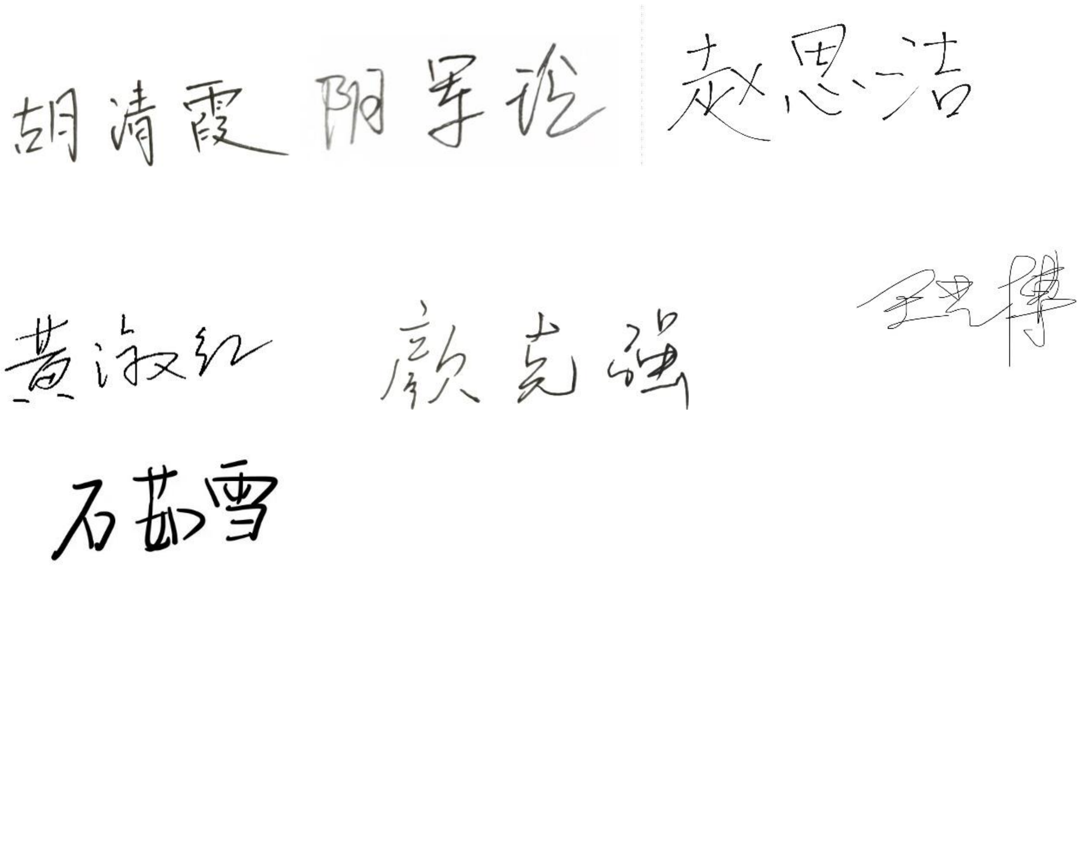

山东第一医科大学附属省立医院涉及人的生物医学研究伦理委员会
伦理审查批件

省医伦批第（NSFC:NO.2020-1322）

项目名称	转录因子 ZFHX3 通过调控 m6A-RNA 甲基化抑制前列腺癌发展的机制研究		
项目负责人	胡清霞	承担科室	临床医学研究院
项目类型	国家自然科学基金项目		
审查文件	1.伦理审查申请；<br>2.研究方案；<br>3.知情同意书；<br>4.其他资料__________		
审查时间	2020 年 3 月 18 日	审查方式	快速审查
伦理审查意见		同意	

审批意见

1.经本伦理委员会审查，同意进行该项目申报。意见和建议：■无　　□有

2.如该项目获得国家自然科学基金委立项，该批件有效；如该项目未获立项，该批件自动失效。

3.伦理委员会对该研究实施过程的年度/定期跟踪审查：■是　　□否

审查期限为研究批准之日起：□三个月　□六个月　■十二个月

4.伦理委员会有权根据实际进展情况改变年度/定期跟踪审查期限。

山东第一医科大学附属省立医院涉及人的生物医学研究伦理委员会

（盖章）

2020 年 3 月 18 日

山东第一医科大学附属省立医院实验动物伦理委员会

批　准　书

批准号：省医动伦批（NSFC:NO. 2020-1111）

项目名称： 转录因子 ZFHX3 通过调控 m6A-RNA 甲基化抑制前列腺癌发展的机制研究

项目负责人：胡清霞　　职称：助理研究员　　联系电话：13652169370

负责研究科室：临床医学研究院

研究起止时间： 2021.01.01-2023.12.31

拟申报项目类别（或在研项目资金来源）：国家自然科学基金青年科学基金项目

评审意见：

研究项目 转录因子 ZFHX3 通过调控 m6A-RNA 甲基化抑制前列腺癌发展的机制研究

经伦理委员会审查：

该项目必须用实验动物进行研究，实验相关人员资格和实验相关单位合适，实验所用动物的品种品系、质量等级、规格基本合适；研究方法符合常规，实验设计基本符合有关动物保护原则、实验动物福利伦理等伦理要求，申请者承诺自觉遵守有关动物实验伦理、随时接受本委员会的监督与检查。

同意开展该项目的研究。

山东第一医科大学附属省立医院实验动物伦理委员会

2020 年　3 月　18 日

Cell Line Authentication Report

Cell Line Authentication Report

Customer: Qingxia Hu

Quotation Number: 80-1481451309_R1

Completion Date: 04/03/2024

1. **Sample ID: LNCap**
2. **Original Material: LNCap**
3. **Methods:**

1).Genomic DNA was extracted from the cell pellets provided by the customer.

2).Samples, together with positive and negative control were amplified using GenePrint 10 System (Promega).

3). Amplified products were processed using the ABI3730xl Genetic Analyzer.

4). Data were analyzed using GeneMapper4.0 software and then compared with the Cellosaurus, ATCC, DSMZ, JCRB and RIKEN databases for reference matching.

4. **Results:**

1) **10 Loci STR Profile：**

Genetic Site	Cell Bank information		Customer sample	
(Locus)	LNCaP		LNCap	
Amelogenin	X	Y	X	Y
CSF1PO	10	11	10	11
D13S317	10	12	10	12
D16S539	11	11	11	11
D5S818	11	12	11	12
D7S820	9.1	10.3	9.1	10.3
TH01	9	9	9	9
TPOX	8	9	8	9
vWA	16	18	16	18
D21S11			29	32.2
Percent match between the sample and the database profile： 100%				

Summary:

Your cell line is considered to be “identical” to the reference cell line in the Cell Bank STR database, as the STR profile yields a 100% match.

Notes：

1. P=100% x (2xM)/N; M=18, N=36 P=100% x (2x18)/36=100%

M: number of the matching peaks; N: number of all peaks

2. Based on the ANSI Standard, cell lines with ≥80% match are considered to be related; i.e., derived from a common ancestry. Cell lines with between a 55% to 80% match require further profiling for authentication of relatedness.

3. The short tandem repeat (STR) profile generated by Azenta is indicative only of the sample sent to Azenta at the time it was sent. This data and analysis are for research use only.

2) Electrophoretogram

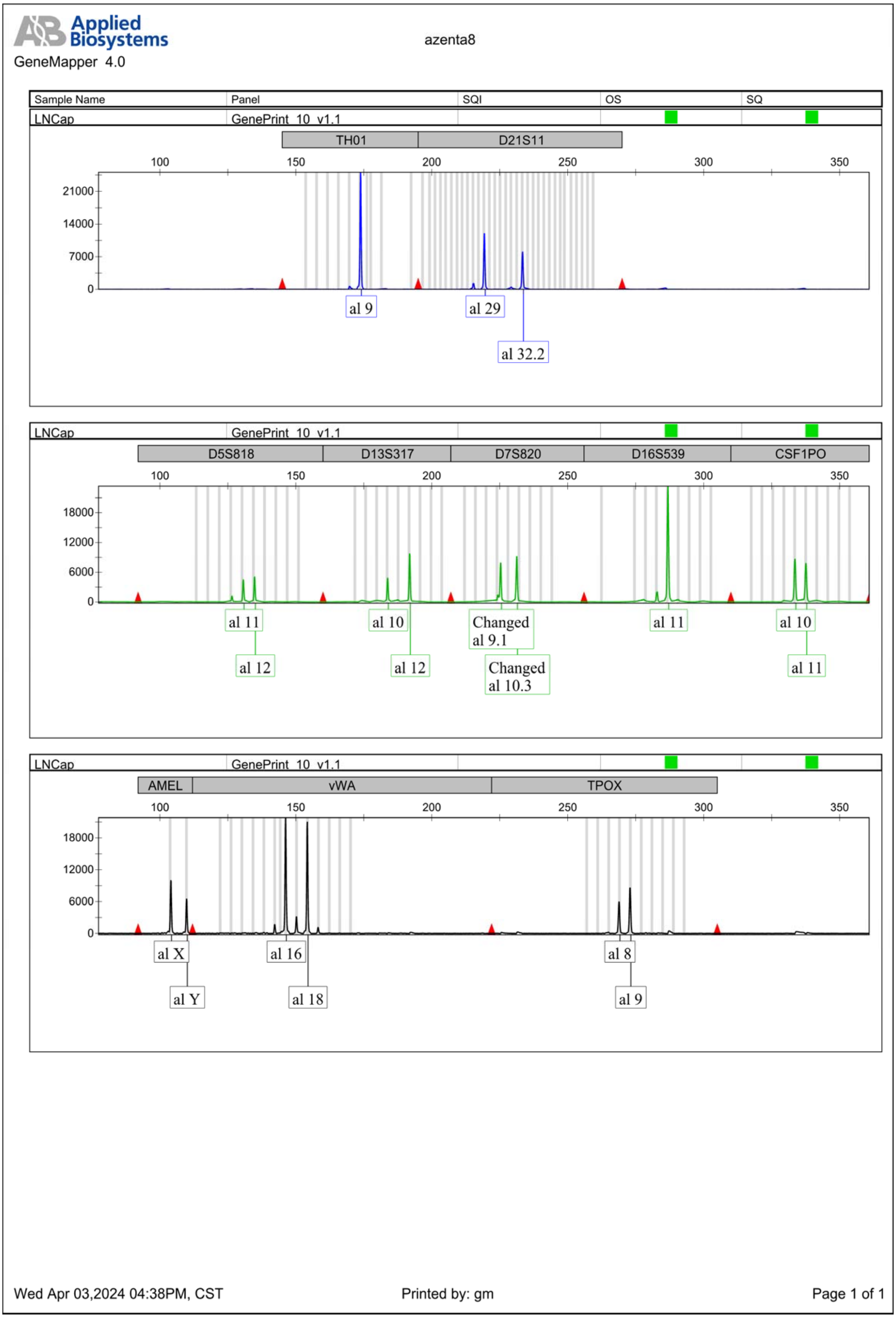

Note：Raw data in appendix

Cell Line Authentication Report

Cell Line Authentication Report

Customer: Qingxia Hu

Quotation Number: 80-1481451309_R1

Completion Date: 04/03/2024

1. **Sample ID: RWPE-1**
2. **Original Material: RWPE-1**
3. **Methods:**

1).Genomic DNA was extracted from the cell pellets provided by the customer.

2).Samples, together with positive and negative control were amplified using GenePrint 10 System (Promega).

3). Amplified products were processed using the ABI3730xl Genetic Analyzer.

4). Data were analyzed using GeneMapper4.0 software and then compared with the Cellosaurus, ATCC, DSMZ, JCRB and RIKEN databases for reference matching.

4. **Results:**

1) 10 Loci STR Profile：

Genetic Site	Cell Bank information		Customer sample	
(Locus)	RWPE-1		RWPE-1	
Amelogenin	X	Y	X	X
CSF1PO	13	13	13	13
D13S317	8	14	8	14
D16S539	9	11	9	11
D5S818	12	15	12	15
D7S820	10	11	10	11
TH01	8	9.3	8	9.3
TPOX	8	11	8	11
vWA	14	18	14	18
D21S11			29	31
Percent match between the sample and the database profile：94%				

Summary:

Your cell line is considered “related” to the reference cell line in the Cell Bank STR database, as the STR profile yields matches that are ≥ 80% but less than 100%.

Notes：

1. P=100% x (2xM)/N; M=17, N=36 P=100% x (2x17)/36=94%

M: number of the matching peaks; N: number of all peaks

2. Based on the ANSI Standard, cell lines with ⩾80% match are considered to be related; i.e., derived from a common ancestry. Cell lines with between a 55% to 80% match require further profiling for authentication of relatedness.

3. The short tandem repeat (STR) profile generated by Azenta is indicative only of the sample sent to Azenta at the time it was sent. This data and analysis are for research use only.

2) Electrophoretogram

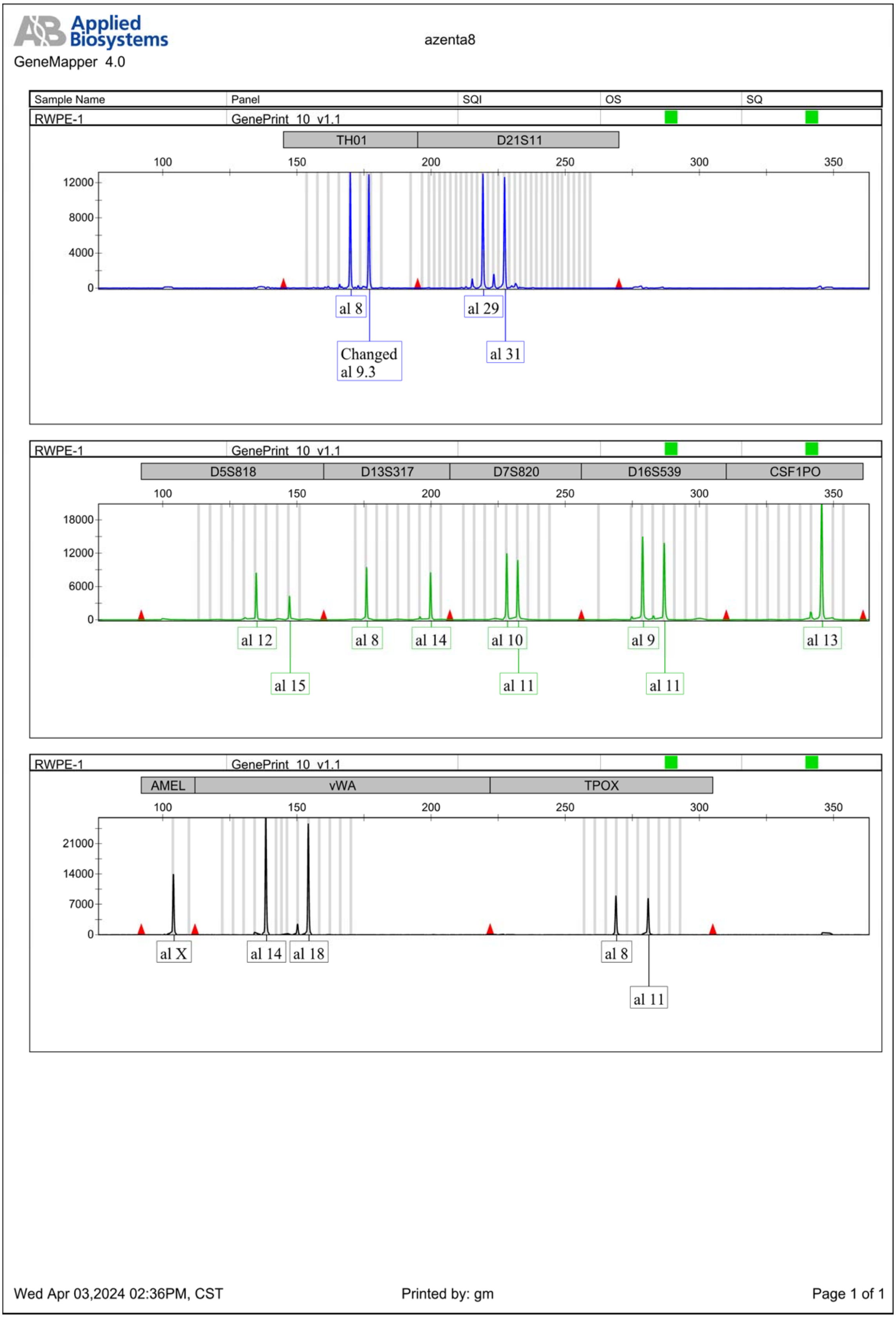

Note：Raw data in appendix

Cell Line Authentication Report

Cell Line Authentication Report

Customer: Qingxia Hu

Quotation Number: 80-1335150457_R2

Completion Date: 10/17/2023

1. **Sample ID: PC-3**
2. **Original Material: PC-3**
3. **Methods:**

   1).Genomic DNA was extracted from the cell pellets provided by the customer.

   2).Samples, together with positive and negative control were amplified using GenePrint 10 System (Promega).

   3). Amplified products were processed using the ABI3730xl Genetic Analyzer.

   4). Data were analyzed using GeneMapper4.0 software and then compared with the Cellosaurus, ATCC, DSMZ, JCRB and RIKEN databases for reference matching.

4. **Results:**

   1) **10 Loci STR Profile：**

Genetic Site	Cell Bank information		Customer sample	
(Locus)	PC-3		PC-3	
Amelogenin	X	X	X	X
CSF1PO	11	11	11	11
D13S317	11	11	11	11
D16S539	11	11	11	11
D5S818	13	13	13	13
D7S820	8	11	8	11
THO1	6	7	6	7
TPOX	8	9	8	9
vWA	17	17	17	17
D21S11			29	31.2
Percent match between the sample and the database profile：100%				

Summary:

Your cell line is considered to be “identical” to the reference cell line in the Cell Bank STR database, as the STR profile yields a 100% match.

Notes：

1. P=100% x (2xM)/N; M=18, N=36 P=100% x (2x18)/36=100%

M: number of the matching peaks; N: number of all peaks

2. Based on the ANSI Standard, cell lines with ≥80% match are considered to be related; i.e., derived from a common ancestry. Cell lines with between a 55% to 80% match require further profiling for authentication of relatedness.

3. The short tandem repeat (STR) profile generated by Azenta is indicative only of the sample sent to Azenta at the time it was sent. This data and analysis are for research use only.

2) Electrophoretogram

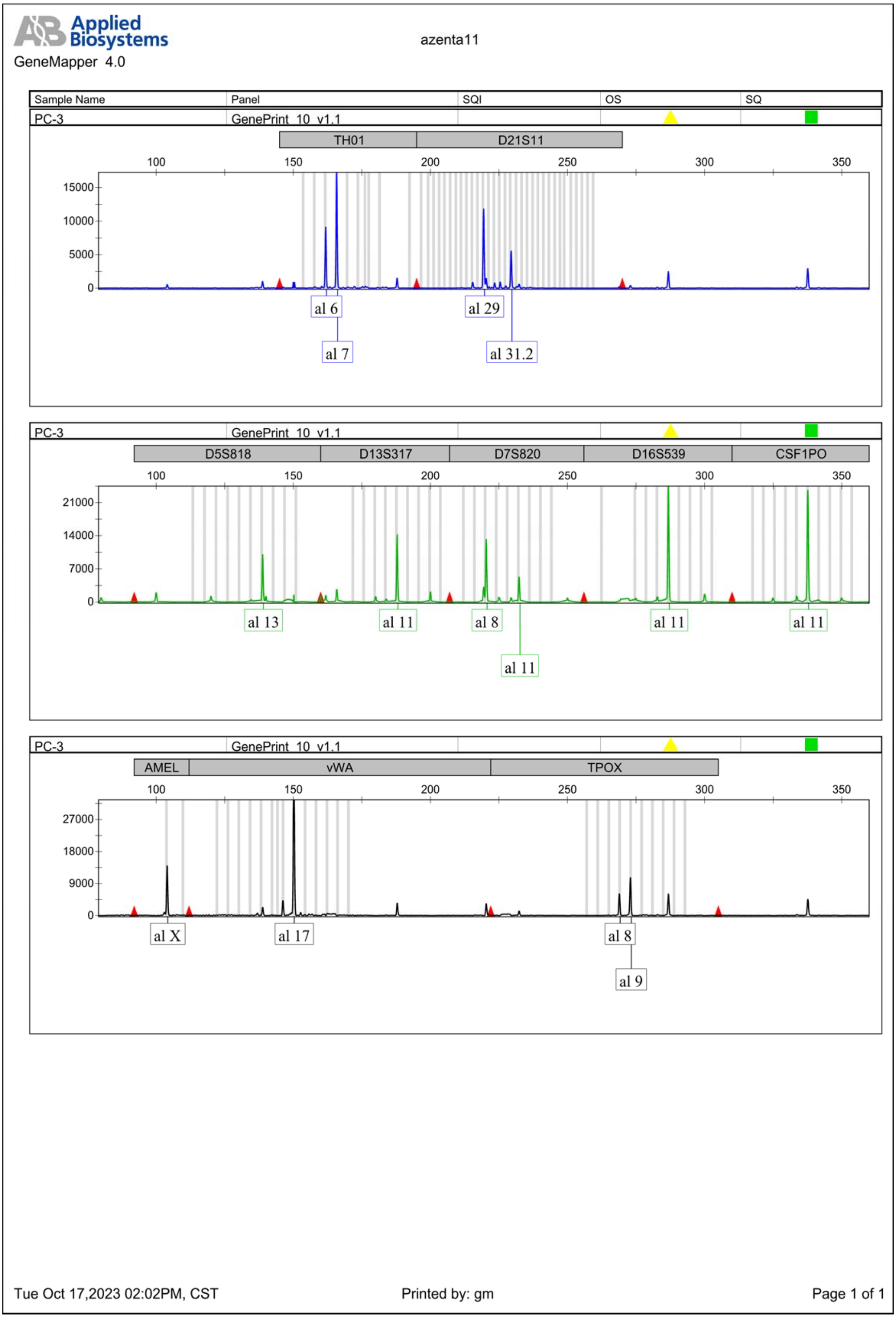

Note：Raw data in appendix

Cell Line Authentication Report

Cell Line Authentication Report

Customer: Qingxia Hu

Quotation Number: 80-1335150457_R2

Completion Date: 10/17/2023

1. **Sample ID: DU-145**
2. **Original Material: DU-145**
3. **Methods:**

1).Genomic DNA was extracted from the cell pellets provided by the customer.

2).Samples, together with positive and negative control were amplified using GenePrint 10 System (Promega).

3). Amplified products were processed using the ABI3730xl Genetic Analyzer.

4). Data were analyzed using GeneMapper4.0 software and then compared with the Cellosaurus, ATCC, DSMZ, JCRB and RIKEN databases for reference matching.

4. **Results:**

1) **10 Loci STR Profile：**

Genetic Site	Cell Bank information		Customer sample	
(Locus)	DU145		DU-145	
Amelogenin	X	Y	X	Y
CSF1PO	10	11	10	11
D13S317	12	14	12	14
D16S539	11	13	11	13
D5S818	10	13	10	13
D7S820	7	10,11	7	10,11
THO1	7	7	7	7
TPOX	11	11	11	11
vWA	17	18,19	17	18,19
D21S11			30	33
Percent match between the sample and the database profile：100%				

Summary:

Your cell line is considered to be “identical” to the reference cell line in the Cell Bank STR database, as the STR profile yields a 100% match.

Notes：

1. P=100% x (2xM)/N; M=20, N=40 P=100% x (2x20)/40=100%

M: number of the matching peaks;   N: number of all peaks

2. Based on the ANSI Standard, cell lines with ≥80% match are considered to be related; i.e., derived from a common ancestry. Cell lines with between a 55% to 80% match require further profiling for authentication of relatedness.

3. The short tandem repeat (STR) profile generated by Azenta is indicative only of the sample sent to Azenta at the time it was sent. This data and analysis are for research use only.

2) Electrophoretogram

Applied Biosystems
GeneMapper 4.0

azenta11

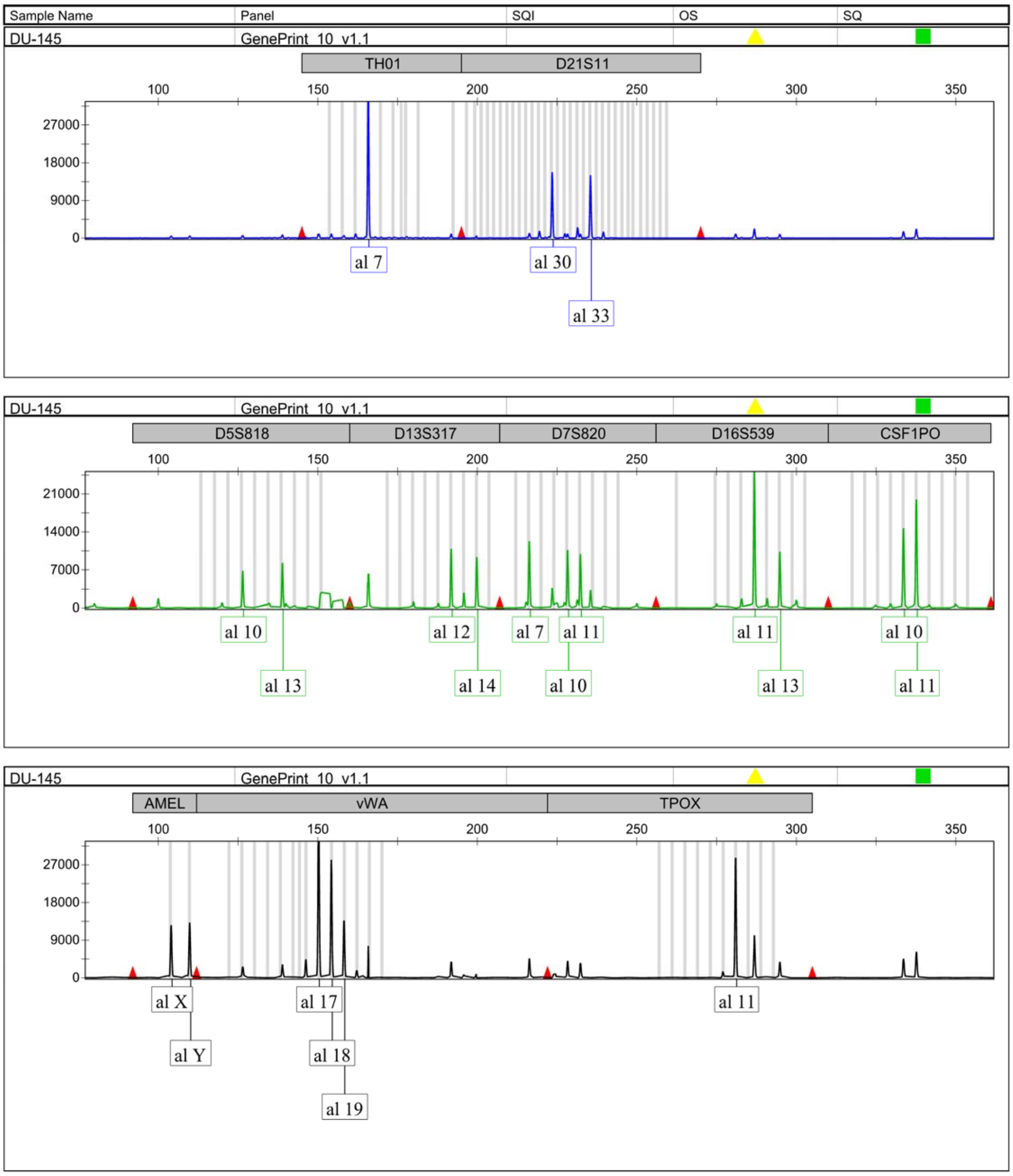

Tue Oct 17,2023 02:00PM, CST

Printed by: gm

Page 1 of 1

Note：Raw data in appendix

Cell Line Authentication Report

Cell Line Authentication Report

Customer: Qingxia Hu

Quotation Number: 80-1335150457_R2

Completion Date: 10/17/2023

1. **Sample ID: 22Rv1**
2. **Original Material: 22Rv1**
3. **Methods:**

1).Genomic DNA was extracted from the cell pellets provided by the customer.

2).Samples, together with positive and negative control were amplified using GenePrint 10 System (Promega).

3). Amplified products were processed using the ABI3730xl Genetic Analyzer.

4). Data were analyzed using GeneMapper4.0 software and then compared with the Cellosaurus, ATCC, DSMZ, JCRB and RIKEN databases for reference matching.

4. **Results:**

1) **10 Loci STR Profile：**

Genetic Site	Cell Bank information		Customer sample	
(Locus)	22Rv1		22Rv1	
Amelogenin	X	Y	X	Y
CSF1PO	10	11	10	11
D13S317	9	12	9	12
D16S539	12	12	12	12
D5S818	11	13	11	13
D7S820	9	10,11	9	10,11
THO1	6	9.3	6	9.3
TPOX	8	8	8	8
vWA	15	21	15	21
D21S11			30	30
Percent match between the sample and the database profile：100%				

Summary:

Your cell line is considered to be "identical" to the reference cell line in the Cell Bank STR database, as the STR profile yields a 100% match.

Notes：

1. P=100% x (2xM)/N; M=19, N=38 P=100% x (2x19)/38=100%

M: number of the matching peaks;   N: number of all peaks

2. Based on the ANSI Standard, cell lines with ≥80% match are considered to be related; i.e., derived from a common ancestry. Cell lines with between a 55% to 80% match require further profiling for authentication of relatedness.

3. The short tandem repeat (STR) profile generated by Azenta is indicative only of the sample sent to Azenta at the time it was sent. This data and analysis are for research use only.

2) Electrophoretogram

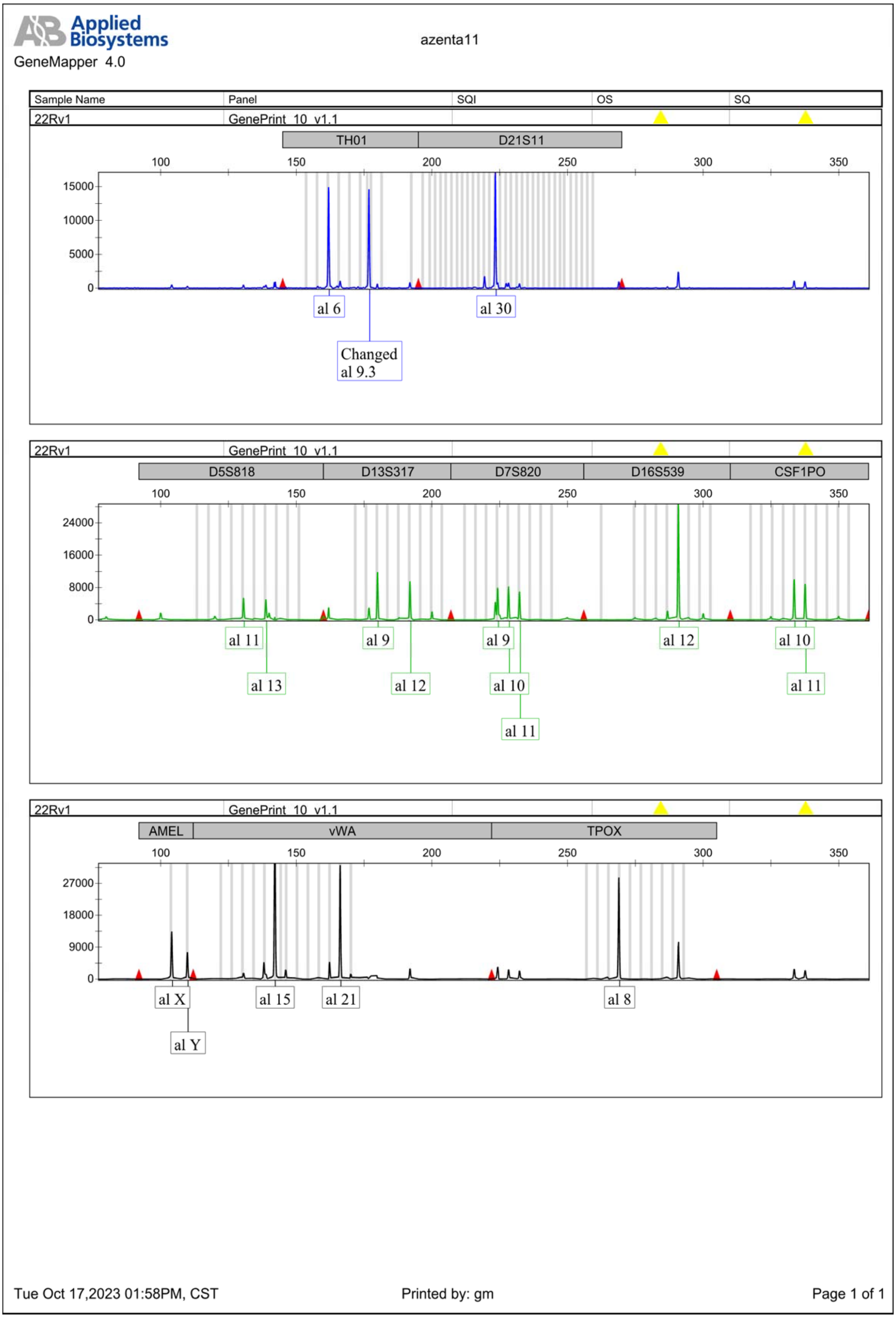

Note：Raw data in appendix
